# Gene gravity-like algorithm for disease gene prediction based on phenotype-specific network

**DOI:** 10.1186/s12918-017-0519-9

**Published:** 2017-12-06

**Authors:** Limei Lin, Tinghong Yang, Ling Fang, Jian Yang, Fan Yang, Jing Zhao

**Affiliations:** 1 Department of Mathematics, Army Logistics University of PLA, Chongqing, China; 2 School of Pharmacy, Second Military Medical University, Shanghai, China; 3 Institute of Interdisciplinary Complex Research, Shanghai University of Traditional Chinese Medicine, Shanghai, China

**Keywords:** Disease gene prediction, Phenotype similarity, Topological similarity, Functional similarity, Gene gravity-like algorithm

## Abstract

**Background:**

Polygenic diseases are usually caused by the dysfunction of multiple genes. Unravelling such disease genes is crucial to fully understand the genetic landscape of diseases on molecular level. With the advent of ‘omic’ data era, network-based methods have prominently boosted disease gene discovery. However, how to make better use of different types of data for the prediction of disease genes remains a challenge.

**Results:**

In this study, we improved the performance of disease gene prediction by integrating the similarity of disease phenotype, biological function and network topology. First, for each phenotype, a phenotype-specific network was specially constructed by mapping phenotype similarity information of given phenotype onto the protein-protein interaction (PPI) network. Then, we developed a gene gravity-like algorithm, to score candidate genes based on not only topological similarity but also functional similarity. We tested the proposed network and algorithm by conducting leave-one-out and leave-10%-out cross validation and compared them with state-of-art algorithms. The results showed a preference to phenotype-specific network as well as gene gravity-like algorithm. At last, we tested the predicting capacity of proposed algorithms by test gene set derived from the DisGeNET database. Also, potential disease genes of three polygenic diseases, obesity, prostate cancer and lung cancer, were predicted by proposed methods. We found that the predicted disease genes are highly consistent with literature and database evidence.

**Conclusions:**

The good performance of phenotype-specific networks indicates that phenotype similarity information has positive effect on the prediction of disease genes. The proposed gene gravity-like algorithm outperforms the algorithm of Random Walk with Restart (RWR), implicating its predicting capacity by combing topological similarity with functional similarity. Our work will give an insight to the discovery of disease genes by fusing multiple similarities of genes and diseases.

**Electronic supplementary material:**

The online version of this article (10.1186/s12918-017-0519-9) contains supplementary material, which is available to authorized users.

## Background

Pinpointing disease genes is a fundamental task in elucidating the pathogenesis of diseases. It has significant implication in disease modeling, drug design, therapeutic prevention and clinical treatment [1]. Disease gene prediction is a process to pick out the most susceptible genes among a pool of candidate genes for further downstream screening.

Traditional disease gene prediction methods involve linkage analysis and genome-wide association study (GAWS). They typically identify a chromosome interval of 0.5~10 *CM*, which includes hundreds of candidate genes [2]. Although such methods have achieved fruitful success in the low-throughput period, they suffer from high false negatives for merely focusing on limited candidates on certain interval of chromosome. Moreover, experimental validation for hundreds of candidates is time-consuming and expensive. Therefore, computational methods are required to accelerate the discovery of disease genes.

With the advent of bioinformatics and the rapid development of high-throughput mapping technology [3], network-based methods arise and boost the discovery of disease associated genes [4]. In general, network-based methods predict potential disease genes based on guilt-by-association principle, in which candidates are more likely to be disease genes if they have higher topological similarity to known disease genes in the background PPI network [5, 6]. Such topological similarity between candidates and known disease genes can be measured from local or global perspectives. The local approaches mainly consider local network topology to infer potential disease genes. Linghu et al. used neighborhood-weighing rule to score candidates based on their linkage weight with the known disease genes [7]. Krauthammer et al. used the shortest distance method to predict disease genes that may not be physically related but belong to common pathways [8]. However, the local approaches always suffer from noisy and incomplete background network and fail in predicting precision [9]. Global methods like RWR [10], network propagation [11] and kernel diffusion [10] have partly solved this problem by considering multiple alternate paths and the global topology of PPI network. Although the global network-based methods outperform the local ones, they still have limitation in disease gene prediction for only considering topological similarity but ignoring other functional information.

Recent years, the importance of phenotype similarity information has attracted community attention and been integrated in network-based methods to identify disease genes. So far, the integration of phenotype similarity information with gene-gene network has been applied mainly in two ways. The first class of methods such as Vavine [12], Prince [11], and Prosim [13], regards known disease genes of similar phenotypes as known disease genes for the given phenotype, so as to enlarge the seed set, which is a collection of known disease genes. This type of application provides alternatives for phenotypes with few known disease genes, but fails to fully exploit the similarity information. The second class of methods, such as Cipher [14], RWRH [15], pgWalk [16] and MAXIF [17], combines phenotype-phenotype similarities, gene-phenotype relations and gene-gene interactions to construct a heterogeneous network. Based on the heterogeneous network, new gene-phenotype relationships are predicted by algorithms. Reasonable as it seems, this type of methods ignores the great difference in gene network and phenotype network, which are comparable neither in biological property nor order of magnitude. Therefore, it remains a challenge for us to utilize phenotype similarity in a more reasonable way.

In this paper, we tried to improve disease gene prediction by integrating the similarity of disease phenotypes, biological functions and network topologies. To achieve this, we first proposed a new way to project phenotype similarity information into the background PPI network and constructed a phenotype-specific network. This new network is tailored to each phenotype and more relevant to the phenotype than the original network. Next, we proposed a gene gravity-like algorithm based on Newton’s law of universal gravitation. The new algorithm is designed to select the potential disease genes which have higher topological similarity measured by RWR algorithm as well as functional similarity measured by the number of common GO terms. In this way, we successfully integrated three types of similarity information to predict new disease genes. We further conducted leave-one-out and leave-10%-out cross validations to assess the performance of the proposed algorithms. At last, the predicting power of the proposed methods was demonstrated by uncovering the test genes in the DisGeNET database. Meanwhile, we went on case study on three complex diseases, namely obesity, prostate cancer and lung cancer.

## Methods

### Data preparation

Our research needs to use PPI data, phenotype similarity data, gene ontology data and disease gene set. These data are extracted from public databases described as follows.

### PPI data

The PPI data used in this paper comes from the HumanNet database. HuamnNet is a functional gene association network that incorporates 21 kinds of ‘omics’ data and assigns confidence of interactions with log-likelihood scores [18]. In the PPI network, proteins encoded by genes are represented by nodes, and interactions are edges with confidence scores, which indicate the likelihood of pairwise genes interacting with each other. In this work, to successfully run global algorithm on the network, we further extracted the biggest connected cluster from the PPI network after removing self-looped and duplicated edges. Finally the PPI network comprises 16,222 genes and 476,388 edges, whose adjacent matrix is 16222 × 16222dimension. In this paper, we still call this final PPI network as HumanNet network or the original network.

### Phenotype similarity data

The phenotype similarity data was downloaded from MimMiner database (http://www.cmbi.ru.nl/MimMiner/suppl.html) created by Van Direl et al. They utilized text-mining method to describe phenotypes by medical subject headings vocabulary (MeSH), and profiled them into corresponding feature vectors. At last a 5080 × 5080 dimensional similarity matrix is obtained by computing the cosine of the angle between pairwise feature vectors [19].

### Gene ontology (GO)

Gene Ontology (GO) is a hierarchical and maintained database that uses controlled vocabulary of terms to annotate genes and their products. GO database develops three structured ontologies from different biological aspects, namely, biological process, cellular component and molecular function [20]. In the GO database, each GO term represents one concept, and indicates certain biological meaning. A GO term which lies in the deeper level in the term ontology indicates more direct gene function, and the GO terms used to annotate a gene are usually the deepest level that so far has been found. Therefore, if gene pairs share more common annotated GO terms, they are more likely to be functionally related. Based on such observation, we can measure functional similarity between genes by the number of their common GO terms. In particular, to calculate the number of common GO terms between gene pairs, we first downloaded GO database on March.1, 2016, and implemented following steps:

1. Removing genes with less than 3 GO terms;
2. Excluding genes that are absent in the HumanNet network;
3. Intersecting common GO terms of gene pairs.

Note that, since the terms annotating the genes indicate the direct function of genes, we did not consider the parent-child relationship between terms in calculating the overlapping GO terms between genes. If we did so, the number of the common GO terms between genes would be too large to reliably measure the functional similarity between genes.

Finally we got a 16222 × 16222 functional similarity matrix corresponding to the genes in the HumanNet network. In the functional similarity matrix, the element represents the number of common GO terms between genes.

### Disease gene set from OMIM database

In this work, disease genes were collected from Morbid map of the Online Mendelian Inheritance in Man (OMIM) database [21]. We identified 113 disease phenotypes containing 633 disease genes with 503 unique ones (One gene may be shared by several disease phenotypes). The selected disease phenotypes must satisfy following criteria:

1. Being a member of MimMiner database.
2. Having at least 3 disease genes which are included in the HumanNet network.

To evaluate the proposed network and algorithm, we used the 633 genes in the 113 disease phenotypes as seed set to conduct leave-one-out cross validation. Further, we chose 30 diseases from the 113 diseases to perform leave-10%-out cross validation, each of which has at least 6 known disease genes. There are 470 disease genes associated with these 30 diseases (The list of these phenotypes and disease genes is available in the Additional file 1: Table S1).

### Test gene set from the DisGeNET database

DisGeNET is a discovery platform which provides open access to one of the largest collections of genes and variants associated with human diseases. It assigns a confidence score to measure the reliability between gene-phenotype relationships. In this work, we downloaded the curated gene-disease association file (http://www.disgenet.org/web/DisGeNET/menu/downloads) and filtered the gene-phenotype relationship with score higher than 0.4. Finally, for the 113 diseases mentioned above, there are 54 diseases included in DisGeNET. A total of 572 genes of the 54 diseases are included in the DisGeNET but not in the OMIM. These genes are used as test gene set to validate the performance of the proposed algorithms (The list of these phenotypes and disease genes is available in the Additional file 1: Table S2).

### Workflow for the prediction of disease genes

**Fig. 1:**
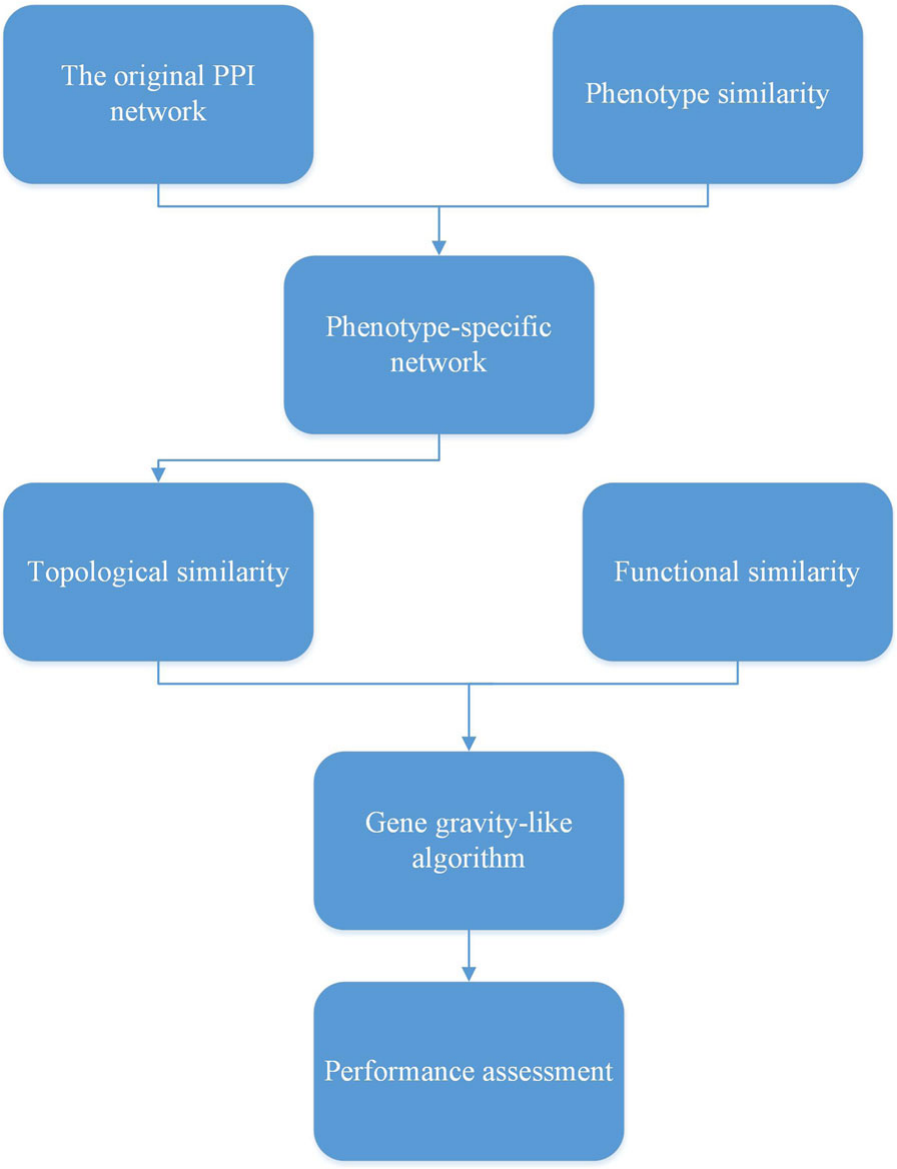
Workflow for the prediction of disease genes

We demonstrate our workflow for the prediction of disease genes in Fig. 1. It is mainly conducted in 3 steps. We start with mapping phenotype similarity information of a given phenotype onto the original PPI network so as to construct a phenotype-specific network. Next, with evidence from both topological distance on the network and functional similarity measured by the number of common GO terms, we score and rank each candidate by gene gravity-like algorithm. At last, we conduct performance assessment to validate proposed network and algorithm.

### Construction of phenotype-specific network

**Fig. 2:**
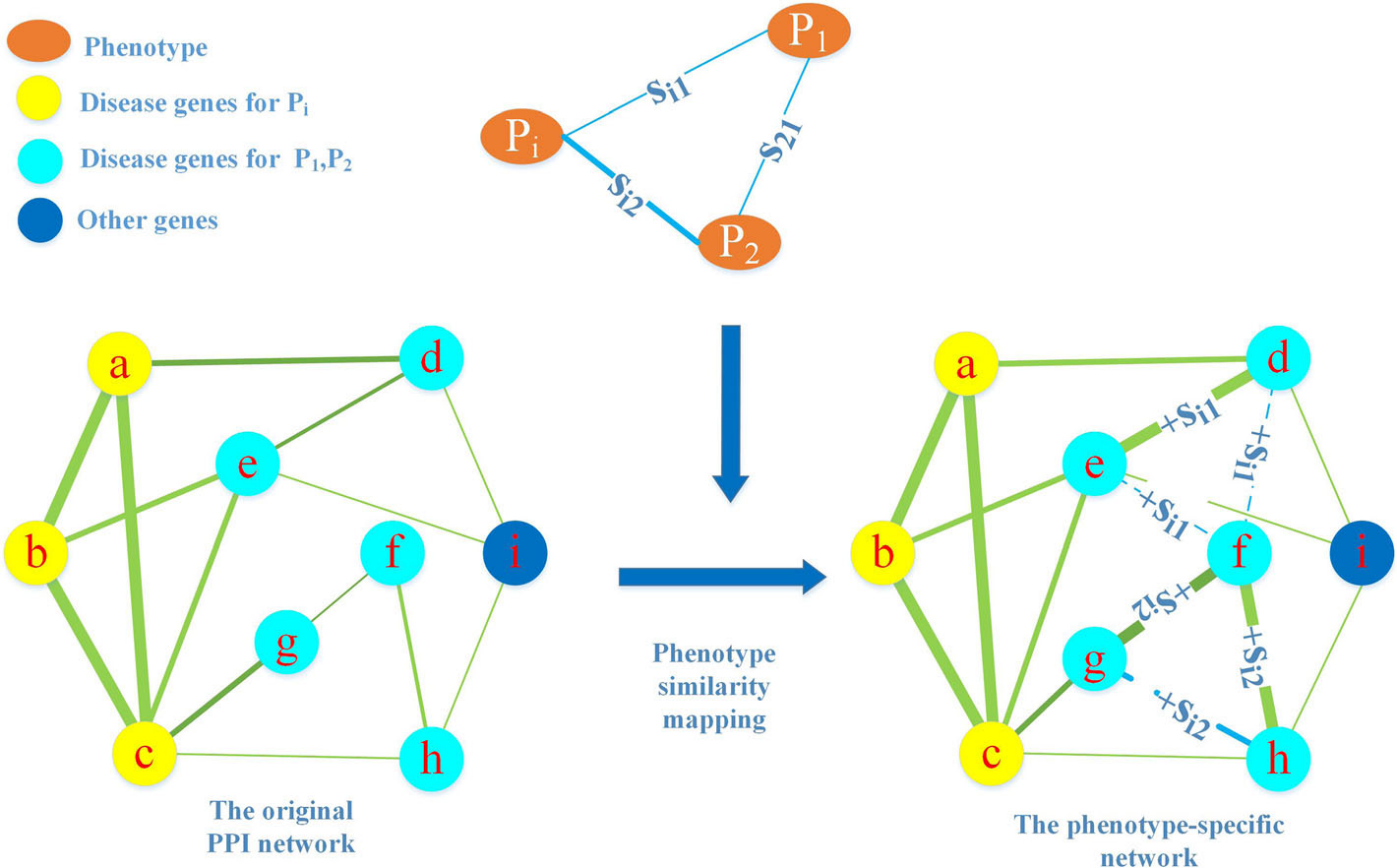
An example for the construction of the phenotype-specific network for phenotype *P*_*i*_ Assume that *P*_1_, *P*_2_ are phenotypes similar to given phenotype *P*_*i*_ with similarity score *s*_*i*1_ and *s*_*i*2_ respectively. {*e*, *d*, *f*} is the disease gene set of *P*_1_, and {*f*, *g*, *h*} is the disease gene set of *P*_2_. The phenotype-specific network is constructed by mapping phenotype similarity information into the original PPI network, in which links between genes of the same phenotype are created while corresponding phenotype similarity score is added to the original weight. The dot line denotes the new generated edge

In order to make the PPI network more informative to the phenotype of interest, we propose a simple but efficient way to incorporate phenotype similarity information into PPI network. Unlike the methods that enlarge seed set or construct a heterogeneous network, we improve disease gene prediction by constructing a particularly designed phenotype-specific network for each phenotype. Specifically, for a given phenotype*P*_*i*_, the adjacent matrix for corresponding phenotype-specific network is defined as follows:

1 $$ {W}^{\left({P}_i\right)}=W+\sum \limits_{\begin{array}{l}j=1\\ {}j\ne i\end{array}}^l{s}_{ij}{A}^{\left({P}_j\right)}i=1,2,\dots, l $$

where ${W}^{\left({P}_i\right)}$ is the adjacent matrix of the phenotype-specific network for *P*_*i*_, *W* is the adjacent matrix of the original PPI network; *s*_*ij*_ is the similarity score between phenotype *P*_*i*_ and*P*_*j*_;${A}^{\left({P}_j\right)}$ is the adjacent matrix of a gene-gene network, which has the same nodes as the PPI network and disease genes of the phenotype *P*_*j*_ (*j* = 1, 2, …, *l*, *j* ≠ *i*) are linked with each other. Its element ${a}_{mn}^{\left({P}_j\right)}$ is defined as:

2 $$ {a}_{mn}^{\left({P}_j\right)}=\left\{\begin{array}{l}1\kern1em m,n\in seeds\left({P}_j\right)\\ {}0\kern1em otherwise\end{array}\right., $$

where *seeds*(*P*_*j*_) is the disease gene set for phenotype *P*_*j*_. In details, if both gene *m* and *n* belong to*seeds*(*P*_*j*_), element ${a}_{mn}^{\left({P}_j\right)}$ in ${A}^{\left({P}_j\right)}$ is assigned a value of 1; otherwise, it is 0. In this way, the new network is specific to phenotype *P*_*i*_ and contains all evidence of phenotype similarity for *P*_*i*_ in the PPI network. In Fig. 2, we give an example to illustrate the process of constructing a phenotype-specific network.

### Gene gravity-like algorithm for the prediction of disease genes

Traditionally, Newton’s law of universal gravitation measures the gravitation between two objects by their masses and distance as follows:

3 $$ {G}_{ij}=k\bullet \frac{M_i\bullet {M}_j}{r^2} $$

where *M*_*i*_ and *M*_*j*_ represent the masses of two objects, *r*represents the distance between them, and *k* is the gravitation constant. This equation means that the gravitation of two objects is proportional to the product of their masses and inversely proportional to the square of their distance. Several gravity-like algorithms have been proposed according to the core idea of Newton’s law of universal gravitation and been successfully applied in different fields, like transportation flow [22], population migration [23] and evolution of cancer genomes [24].

In the context of disease gene prediction, we assume that genes having larger interaction force with known disease genes are more likely to be disease genes. Thus we try to use gravitation for the measurement of the interaction force. In the gravitation eq. (3), we take *r* as the topological distance in the background PPI network, *M*_*i*_ as the set of GO terms for gene*i*_,_ and the gravitation constant *k* as 1. The product between *M*_*i*_ and *M*_*j*_ is defined as the number of elements in their intersection set: *M*_*i*_ ∙ *M*_*j*_ = |*M*_*i*_ ∩ *M*_*j*_|. The topological distance between genes is measured by RWR algorithm. In this way, we propose a novel predicting algorithm called gene gravity-like algorithm to score a candidate gene by a set of seed genes. Formally, the equation is:

4 $$ {G}_m^{\left({P}_i\right)}=\frac{{\left(\sum \limits_{n\in seeds\left({P}_i\right)}\left|{M}_m\cap {M}_n\right|\right)}^{\alpha }}{{\left(\sum \limits_{n\in seeds\left({P}_i\right)}\left(1/{R}_{mn}^{RWR}\right)\right)}^{\beta }} $$

where *P*_*i*_ denotes the phenotype of interest and *seeds*(*P*_*i*_) is its seed set; gene *m* is a candidate gene and gene *n* is one of seed genes; |*M*_*m*_ ∩ *M*_*n*_| represents the number of common GO terms shared by gene pair (*m*, *n*); *α* and *β* are parameters that control the contribution of masses and distance respectively. ${R}_{mn}^{RWR}$ stands for the probability that a random walker starting from seed node *n* reaches candidate node *m* in the steady state of a RWR process on the background network. Note that ${R}_{mn}^{RWR}$ is probability which is inversely proportional to distance, thus topological distance between nodes *m* and *n* is measured by$\frac{1}{R_{mn}^{RWR}}$.

The value of ${R}_{mn}^{RWR}$ is calculated by RWR algorithm, a widely applied method that captures overall topological property of the network. The algorithm mimics a random walker who sets out from a seed node, and at each moment chooses to either reach its neighboring node with a rate proportional to the edge weight, or return back to the seed node with a restart probability. The random process can be depicted as follows:

5 $$ {x}^{t+1}=\left(1-c\right){W}_{RW}{x}^t+{cx}^0 $$

6 $$ {W}_{RW}\left(u,v\right)=w(uv)/W(u) $$

where *W*_*RW*_ is the transition matrix obtained by column-normalizing the adjacent matrix *W*, as shown in eq. (6); *x* ^0^ is the initial vector, which is constructed such that equal probabilities are assigned to the seed nodes and sum up to 1; *x* ^*t*^ is the vector whose *i* ‐ *th* element holds the chance of the walker arriving at node *i* at the moment *t*; *c* denotes the restart probability. RWR process is a finite Markov chain. Since finite Markov chain in connected non-bipartite graph guarantees to reach steady state, when the walker walks iteratively in sufficient time, we can get the final probability vector*x* ^∞^. Usually, the steady state is obtained when |*x* ^*t* + 1^ − *x* ^*t*^| < *η* (*η* is a rather small value) [25].

Finally, candidates are scored by eq. (4) and ranked in a descending order. The top ranked genes above certain cutoff are predicted as disease genes of the phenotype under study.

### Performance assessment of the proposed algorithms

To estimate the predicting capacity of a method, we conduct leave-*k*-out cross validation for all disease genes. In each round of validation, *k* genes are randomly removed from the seed set and termed as *test genes*. Next, the *test genes* are ranked together with other candidates based on their scores calculated by the left seed genes [26].

In principle, seed set is composed of all known disease genes, and candidate set can either be the whole genome in the PPI network or the chromosomally nearest 100 genes of the *test genes*. In this work, we take the whole genome as candidates and the disease genes extracted from the OMIM database as seed set. Performance assessment are conducted by leave-one-out and leave-10%-out cross validations. In detail, leave-10%-out cross validation is to take out 10% seed genes as *test genes* and the left seed genes serve as seed set in each round of validation.

After obtaining the ranks of all *test genes* in the leave-*k*-out cross validation, we can systematically compare different methods by following evaluation criteria:

1. Compare the number of disease genes which are ranked above top K. This criterion attaches greater importance to precision. Given that computational method is for efficiently narrowing down experimental screening, only top K genes actually matter for downstream work.
2. Pool together all *test genes*’ rank and calculate the fraction of disease genes by varying rank cut off in the interval $\left[0,\kern0.5em 100\right]$.
3. Plot ROC curve and compute AUC value. ROC (false positive rate vs true positive rate) curve is plotted by thresholding the rank cutoff from 1 to 100. In detail, false positive rate is the fraction of non-seed genes ranked above the threshold, while true positive rate is the proportion of seed genes ranked above the threshold. AUC is the area under the ROC curve, which lies in the interval [0.5, 1]. It will be 0.5 if all disease genes are distributed at random in the rank, and larger area indicates better performance [27].

Note that, since some undiscovered true disease genes are defined as false positives in the validation, those criteria may underestimate the actual performance.

## Results and discussion

In this section, we started out the discussion by evaluating the performance of phenotype-specific networks in contrast with the original PPI network and heterogeneous network. Then, based on phenotype-specific network, we tested whether gene gravity-like algorithm outperforms RWR algorithm. Next, we investigated the influence of parameters in eq. (4) and eq. (5). Further, we compared the performance of gene gravity-like algorithm and RWR algorithm on the two types of networks when it comes to predict the test genes from the DisGeNET database. At last, we employed proposed network and algorithm to predict disease genes for obesity, prostate cancer and lung cancer, and manually checked whether the prediction results are supported by literature or database evidence.

### Performance of phenotype-specific network

To validate the improvement of phenotype-specific networks for the prediction of disease genes, we compared the new networks with the original network by RWR algorithm and the heterogeneous network by RWRH algorithm (Random Walk with Restart on Heterogeneous Network), respectively. RWRH algorithm is a state-of-art method that utilizes phenotype similarity information to detect disease genes. In essence, RWRH is an application of RWR algorithm on a heterogeneous network, which is constructed by connecting the gene network and phenotype network using the gene-phenotype bipartite graph.

In this section, we employed RWR algorithm to conduct disease gene prediction based on the three types of networks respectively. Leave-one-out and leave-10%-out cross validation were used to compare the performance of different types of background networks.

In the leave-one-out cross validation, 113 diseases with 633 known disease genes were applied in validation. For the validation of the phenotype-specific networks, we first constructed 113 phenotype-specific networks. Next, we validated each known disease gene based on the phenotype-specific network that it belongs to. Finally, we pooled together the ranks of all disease genes and analyzed the overall performance. For the validation of the original network, each known disease gene is scored by RWR algorithm according to its connectivity with the rest disease genes of a given disease based on the original PPI network (HumanNet). For the validation of the heterogeneous network, we constructed one heterogeneous network with the same data sources as the phenotype-specific networks have. First, the PPI network is the HumanNet network and the phenotype network is constructed from MimMiner database. Then we connected the two networks by gene-phenotype relationship collected from the OMIM database. In each round, a seed gene is taken out for validation and the corresponding gene-phenotype link is removed from the heterogeneous network. The given phenotype and the remaining disease genes of this phenotype are used as seed nodes. At last, we scored all candidate genes by RWRH algorithm (actually RWR algorithm based on this heterogeneous network).

In the leave-10%-out cross validation, we chose 30 diseases with 470 disease genes from the 113 diseases, so as to keep the number of seed genes for each disease larger than 6. In each round of validation, 10% seed genes for the given disease were taken out as *test genes.* If the number is not an integer, we rounded it up. The validation process is similar to that of leave-one-out cross validation.

**Fig. 3:**
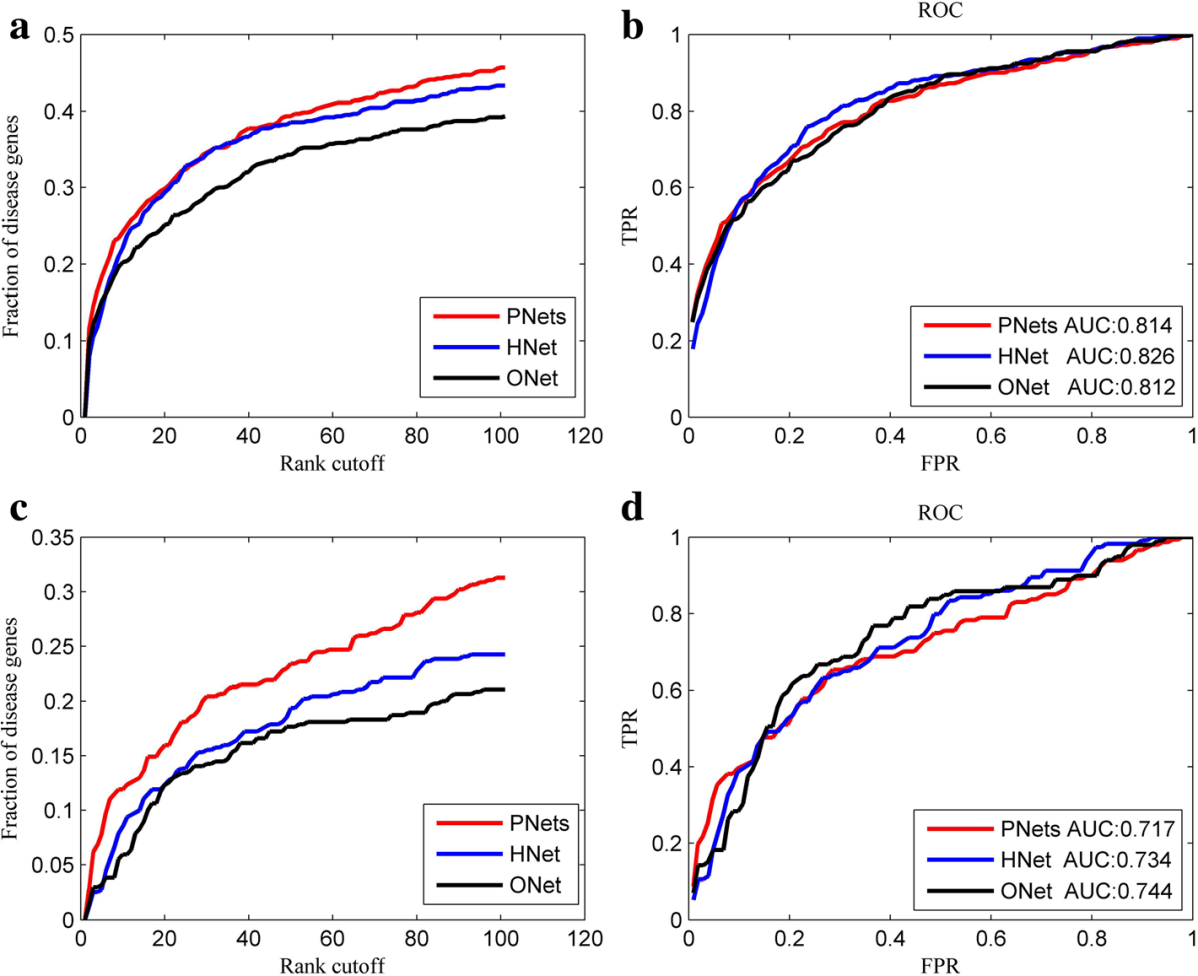
The performance comparison of phenotype-specific networks (PNets), the heterogeneous network (HNet) and the original network (ONet). **a**, **c** fraction of disease genes ranked within top 100 in leave-one-out cross validation and leave-10%-out cross validation, respectively; (**b**), (**d**) ROC curves for the prediction of disease genes in leave-one-out cross validation and leave-10%-out cross validation, respectively

The comparison results are elaborated in Table 1 and Fig. 3. As listed in Table 1, in the leave-one-out cross validation, the phenotype-specific networks outperform the other types of networks in all top K criteria while the original network has the worst performance. Although the heterogeneous network is inferior to the original one in the top 1 criterion (with 49 to 63), it has better overall performance in the other three top K criteria. In detail, in the leave-one-out cross validation (Fig. 3a), there are 46% disease genes ranked within top 100 by phenotype-specific networks, while only 43% and 39% by the heterogeneous network and the original network, respectively. In the leave-10%-cross validation (Fig. 3c), the phenotype-specific networks improve the performance by 10% over the original PPI network. Also, the new networks recover 58 disease genes as top 1 while only 28 by the original one. We further plotted the ROC curve and computed the AUC value for the prediction results based on each type of networks. In Fig. 3b, d, it is observed that the difference of AUC values between the three types of networks is very small. In practice, top K genes are more vital to the identification of novel disease genes. On the whole, the phenotype-specific networks have the highest precision and comparable AUC value. They are seconded by the heterogeneous network which has moderate precision and AUC value. The original network is the weakest in recovering the disease genes.

**Table 1:** Top K comparison for the number of validated disease genes based on phenotype-specific networks, the heterogeneous network and the original network

Rank	Phenotype-specific networks	The heterogeneous network	The original network
Top1	73	49	62
Top5	125	103	102
Top10	158	150	129
Top100	289	274	249

In summary, the validation results suggest that the phenotype-specific networks are more capable of discriminating disease genes among genome than the other two networks. It also validates previous assumption that phenotype similarity information has positive effect on disease gene prioritization. The preferable performance of phenotype-specific network can be ascribed to the reasonable augment of the connectivity among functionally related genes by taking phenotype similarity into account.

### Performance of gene gravity-like algorithm on phenotype-specific network

**Fig. 4:**
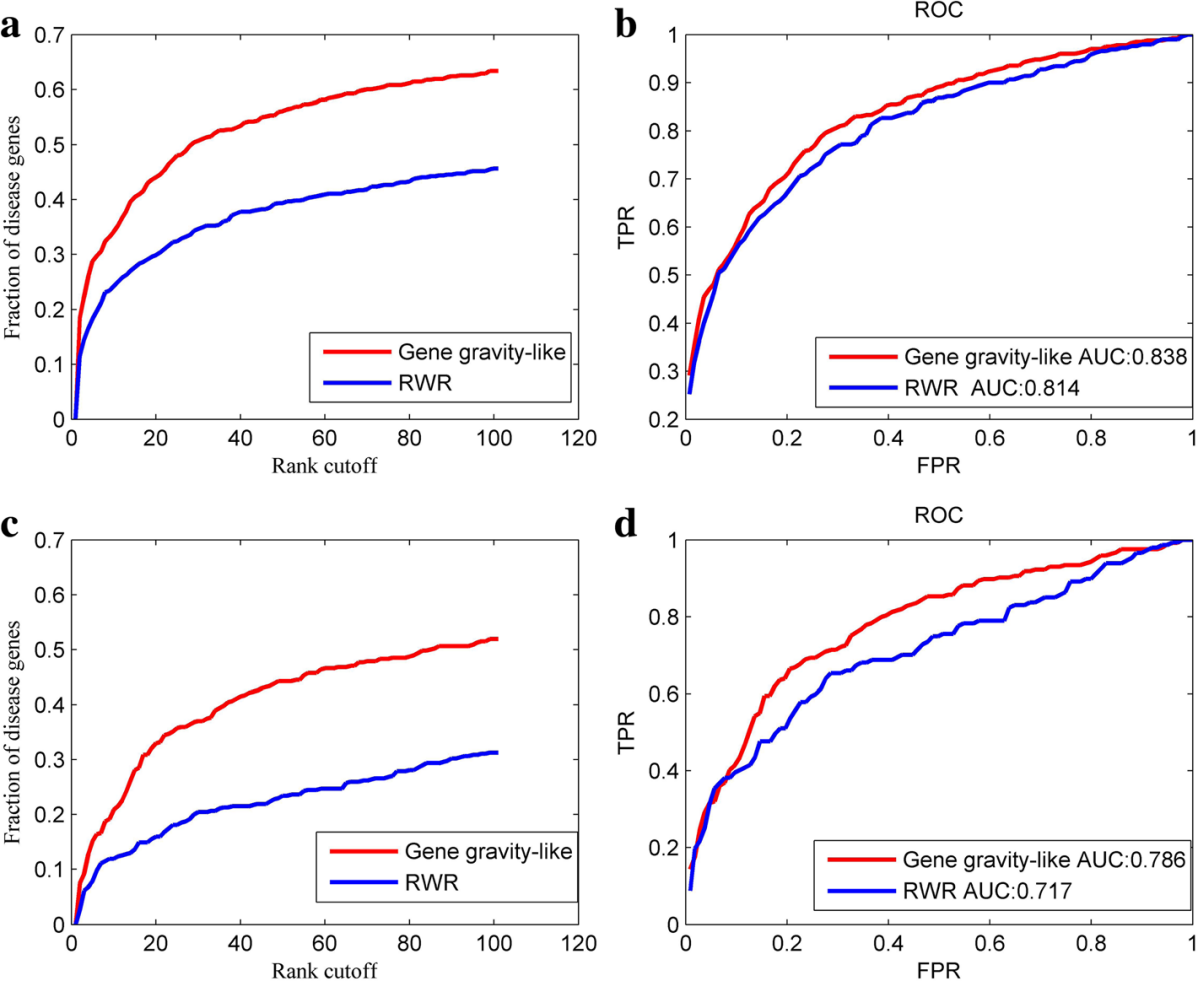
The performance comparison of gene gravity-like algorithm and RWR algorithm. **a**, **c** fraction of disease genes ranked within top 100 in leave-one-out cross validation and leave-10%-out cross validation, respectively; (**b**), (d) ROC curves for the prediction of disease genes by the two algorithms in leave-one-out cross validation and leave-10%-out cross validation, respectively

In this section, we used phenotype-specific networks as background networks to conduct disease gene prediction using the gene gravity-like algorithm and RWR algorithm, respectively. Leave-one-out and leave-10%-out cross validations were applied to compare the two algorithms. Here we set *α* = *β* = 1 in eq. (4). The results are illustrated in Table 2 and Fig. 4. Obviously, in the two kinds of validation, gene gravity-like algorithm outperforms RWR algorithm by a large margin no matter in the aspect of precision (Fig. 4a, c) or AUC value (Fig. 4b, d). Meanwhile, compared with the results of RWRH shown in the last section, the proposed algorithm also does better than RWRH algorithm in both aspects. As shown in Table 2, in the leave-one-out cross validation, the new algorithm predicted 117 true disease genes as top 1, 188 as top 5, 223 as top 10. In contrast, only 73 as top 1, 125 as top 5 and 158 as top 10 were predicted by RWR algorithm. In total, there are 63% disease genes ranked within top 100 by gene gravity-like algorithm while only 46% by the RWR algorithm (Fig. 4a). In the leave-10%-out cross validation, the result is in accordance with that of leave-one-out cross validation (Fig. 4c, d).

**Table 2:** Top K comparison for the number of validated disease genes by gene gravity-like algorithm and RWR algorithm

Rank	Gene gravity-like algorithm	RWR algorithm
Top1	117	73
Top5	188	125
Top10	223	158
Top100	401	289

Consequently, the comparison results indicate that our algorithm is superior to RWR algorithm. The good performance of gene gravity-like algorithm can be attributed to the functional similarity information included in eq. (4), which takes the number of common GO terms between two genes as the product of masses. In addition, we reinforced the importance of topological distance by letting the random walker start from each seed node rather than from all the seed nodes simultaneously. In short, the results support our attempt to use the gravitation equation for the measurement of the interaction force between genes.

### Parameter tuning in the gravity-like algorithm

**Fig. 5:**
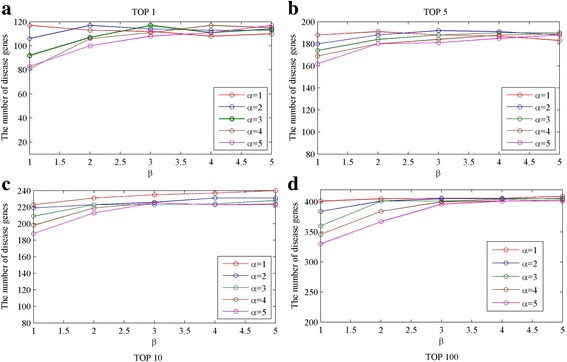
Prediction performance for different combination of α and β**. a** the number of disease genes ranked within top 1; (**b**) the number of disease genes ranked within top 5; (**c**) the number of disease genes ranked within top10; (**d**) the number of disease genes ranked within top100

In the gene gravity-like algorithm, three parameters, namely parameter *c* in Eq. (5), parameters *α* and *β* in Eq. (4), need to be selected. The parameter *c* denotes restart probability in the RWR algorithm. As previous studies have suggested, the value of *c* makes no big difference when ranging in the interval of $\left[0.1,\kern0.22em 0.9\right]$ [10]. In this work, we set it as 0.4. Parameter *α* and *β* control the contribution of mass and distance in the gravity-like equation, respectively. In leave-one-out cross validation, the two parameters were selected from the set{1, 2, 3, 4, 5}. We tuned the two parameters with 25 groups of combination and assessed their performance by top K criteria. The results are depicted in Fig. 5. In most cases, when *α* = 1, the overall performance is better. Moreover, it is observed that there is no obvious fluctuation when *β* is taken from the set{3, 4, 5}, and when *β* = 1, the performance difference of different *α* is most significant. In fact, the prediction results are not very sensitive to the two parameters. Therefore, we took *α* = *β* = 1. This combination has good performance in top K criteria and can reduce the computing complexity.

### Evaluation of new predictions using the DisGeNET database

In this section, we validate the capacity of the gene gravity-like algorithm to predict new disease genes. We extracted 572 genes associated with 54 diseases from the DisGeNET database and used them as test genes. In order to fairly assess the predicting capacity of the proposed algorithms, there is no intersection between the test genes from the DisGeNET database and the known disease genes from the OMIM database. At the same time, the 572 suspectable disease genes are included in the PPI background network.

**Fig. 6:**
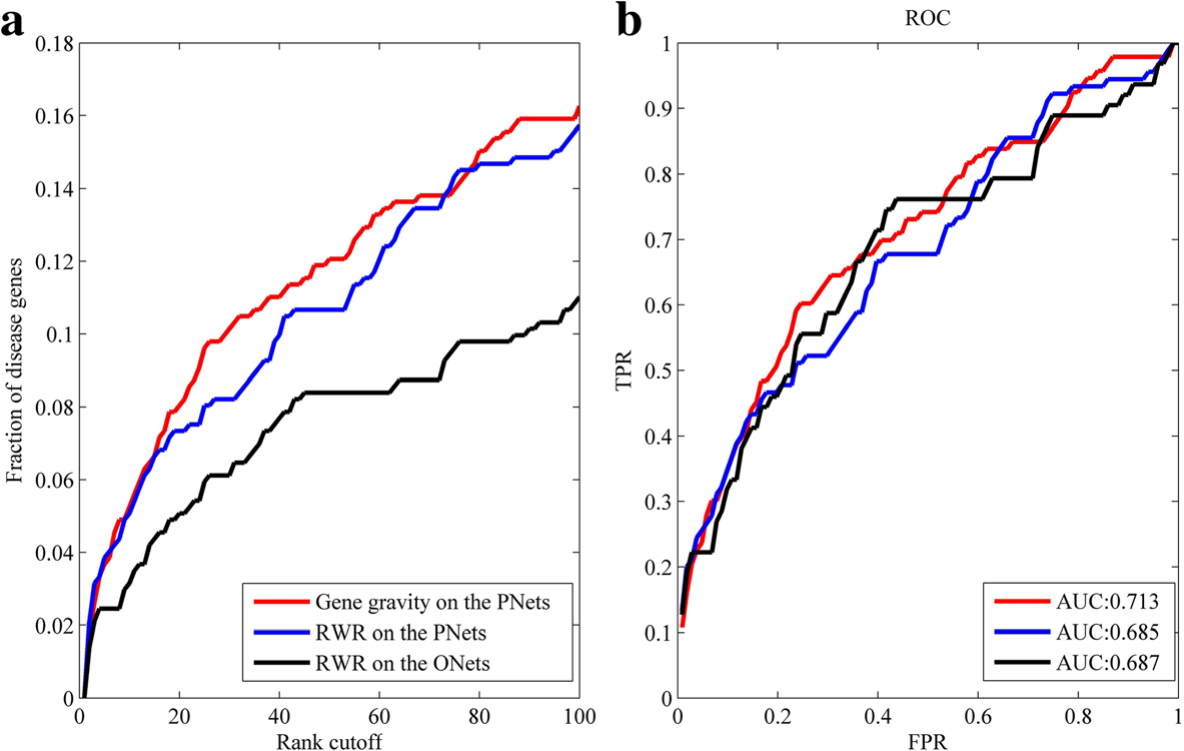
Identification of the test genes in the DisGeNET database by the three algorithms. **a** fraction of the test genes ranked within top 100; (**b**) ROC curves for the prediction of test genes

We took the known disease genes extracted from the OMIM database as the seed set. Then, all candidates (including the 572 disease genes) are ranked based on their connectivity with the seed set. Next, we computed the fraction of the 572 test genes which were ranked within the interval of [0,100]. The predictions were conducted by the three algorithms respectively, namely RWR algorithm on the original network, gene gravity algorithm and RWR algorithm on the phenotype-specific networks. As Fig. 6 indicates, the performance of the gene gravity-like algorithm on the phenotype-specific networks is the best no matter in the number of test genes ranked within top 100 or in the ROC curve. Therefore, the performance of RWR on the new networks is better than that of RWR on the original network in the prediction of new disease genes.

In general, the result in Fig. 6 supports the conclusion made in the previous sections. That is, the new algorithm is superior to the RWR algorithm and the phenotype-specific networks improve the predicting capacity over the original network.

### Case studies: Identifying new disease genes for obesity, prostate cancer and lung cancer

In this section, we tried to predict potential disease genes for obesity, prostate cancer and lung cancer by gene gravity-like algorithm based on the phenotype-specific networks. First, we built three phenotype-specific networks for the three diseases. Then we used their known disease genes in the OMIM database as seed set and employed gene gravity-like algorithm to predict more disease genes. At last, we performed literature or database search to verify the predicted genes. We took the top 20 ranked genes as predicted disease genes and listed the results in Table 3.

**Table 3:** Top 20 predicted disease genes for obesity, prostate cancer and lung cancer

Obesity			Prostate cancer			Lung cancer		
rank	Gene symbol	Class	rank	Gene symbol	Class	rank	Gene symbol	Class
1	ADRB2	√	1	AR	√	1	EGFR	√
2	PPARG	√	2	PTEN	√	2	ERBB2	√
3	ADRB3	√	3	ZFHX3	√	3	BRAF	√
4	MC4R	√	4	BRCA2	√	4	KRAS	√
5	ENPP1	√	5	CDH1	√	5	CASP8	√
6	GHRL	√	6	CHEK2	√	6	PIK3CA	√
7	UCP3	√	7	HIP1	√	7	PARK2	√
8	NR0B2	√	8	MXI1	√	8	FASLG	√
9	POMC	√	9	MAD1L1	√	9	MAP3K8	√
10	CARTPT	√	10	KLF6	√	10	RASSF1	√
11	UCP1	√	11	MSR1	√	11	IRF1	√
12	PPARGC1B	√	12	CD82	√	12	ERCC6	√
13	AGRP	√	13	TP53	*	13	SLC22A18	√
14	SDC3	√	14	BRCA1	*	14	PPP2R1B	√
15	HNF4A	~	15	MAD2L1	~	15	DLEC1	√
16	ESR1	*	16	EGFR	*	16	HRAS	*
17	SIM1	√	17	SIN3A	*	17	AKT1	*
18	MC3R	*	18	MAX	*	18	TP53	*
19	MC1R	*	19	CTNNB1	~	19	GRB2	~
20	LEP	*	20	MYC	*	20	TGFBR2	*

Genes with class mark √ is known disease genes in the OMIM database; * denotes the predicted disease genes with literature or database support; ~ is the predicted genes without evidence

Obesity (MIM: 601,665) is a metabolic disease involving the dysfunction of multiple genes in various biology processes. Over the decades, with energy consumption over energy expenditure, obesity has been one of epidemic diseases that challenge the whole society. However, the genetic mechanism underlying obesity is still ambitious. Here we used the 15 known disease genes in the OMIM database as seeds and ranked candidates over the whole genome. As shown in Table 3, of the top 15 predicted genes, 14 known disease genes are successfully detected, with precision of 93%. For genes that are not included in the OMIM database yet, we tried to verify them with evidence collected from various databases and literatures. ESR1 and MC3R are obesity-associated genes supported by Hancock et al. [28]. The 19th ranked gene MC1R is an important paralog of MC4R, which is a known causal gene for obesity in the OMIM database, and they have similar GO annotations including G-protein coupled receptor activity and hormone binding. In GeneCards database (http://www.genecards.org/), LEP turns out to be the most relevant gene to obesity. Therefore, 19 genes ranked within top 20 are guilty of inducing obesity.

Prostate cancer (MIM: 176,807) is a kind of reproductive disease that varies according to geographic regions and races. Here we constructed a prostate-specific network, and took the 12 prostate-related genes in the OMIM database as seed set. They are successfully ranked within the top 12, with 100% accuracy. The 13th ranked gene TP53 (Tumor Protein P53) encodes a tumor suppressor protein with the function of transcriptional activation, DNA binding, and oligomerization domains. Sung-Gil Chi et al. found that the gene mutations of TP53 are significantly expressed in prostate cancer, indicating the possible involvement of a carcinogenic agent [29]. In addition, when we retrieved the relevant genes for prostate cancer in the GeneCards database, EGFR and BRCA1 are ranked prior to the known causal gene PTEN and BRCA2 respectively. Also CTNNB1, MYC and MAX (MYC Associated Factor X) are judged as causative genes for prostate cancer according to GeneCards database. Finally, 18 of the top 20 genes are associated with prostate cancer.

Lung cancer (MIM: 211,980) is the most common cancer-related death in men and second in woman. It is induced by the rampant cell growth in malignant lung tumor. Lung cancer can be classified into two types: Small cell lung cancer and Non-small-cell lung cancer. We took the 16 known disease genes in the OMIM database as seed nodes and predicted the top 20 ranked genes as disease genes for lung cancer. Among the top 16 of the prediction list, there are 15 known causal genes unraveled as true positives. HRAS, the 16th ranked gene, belongs to the Ras oncogene family. Dysfunction in this gene is implicated in a wide spectrum of cancers. TGFBR2 is a transforming Growth Factor Beta Receptor 2 which may induce Esophageal Cancer. Aforementioned two genes are susceptible to the lung cancer according to GeneCards database. As for AKT1 and TP53, they participate in the Small cell lung cancer pathway according to PathCards database (https://pathcards.genecards.org/). Therefore, 19 genes ranked within top 20 have supportive evidence.

On the whole, the results in Table 3 implicate the capacity of proposed algorithms in capturing novel disease genes. It validates the advantage of our prediction algorithm which integrates the information of phenotype similarity, functional similarity and topological similarity.

## Conclusion

To make better use of phenotype and functional information into the network-based prediction of disease genes, we proposed gene gravity-like algorithm based on phenotype-specific networks. First, for each phenotype we constructed a phenotype-specific network by integrating phenotype similarity information into PPI network. Being used as background network in the prediction of disease genes, the phenotype-specific network shows notably better performance than the original PPI network and the heterogeneous network. It demonstrates the importance to consider phenotype modularity in detecting gene-phenotype relationship. Moreover, compared with the heterogeneous network, our phenotype network projects phenotype information into background network in a more reasonable way. Next, we devised a novel computational model called gene gravity-like algorithm, inspired by Newton’s law of universal gravitation, to identify gene-phenotype relationship. In this algorithm, we employed RWR algorithm to measure the topological distance between seed and candidate, and calculated the number of their common GO terms as the product of their masses. The validation results preferred our algorithm to RWR and RWRH algorithm, which can be ascribed to the augment of topological similarity and the use of functional similarity information from GO database. Moreover, disease genes in the DisGeNET database served as test gene set to validate the better performance of the gene gravity-like algorithm and phenotype-specific network over the RWR algorithm and the original network, respectively. At last, we tested the predictive capacity of the proposed network and algorithm through case studies on the obesity, prostate cancer and lung cancer. Once again, the results proved the superiority of the proposed network and algorithm in real applications. In conclusion, our work could shed new light on the way to integrate the similarity of disease phenotypes, biological functions and network topologies in the prediction of disease genes.

In spite of the good performance of our methods, we suggest that there is still broad space to improve. First, the phenotype similarity information used in this work is rather limited. Actually, Mimniner database has not updated since published. In future, more efforts need to be devoted to digging the similarity of wider spectrum of phenotypes. Second, instead of using GO information in eq. (4), future work could consider to combine more functional similarity information, such as gene co-expression [30, 31] and tissue-specific expression [32]. Third, the quality of PPI network is at the core of disease gene prediction. Although there have been several methods that focus on integrating heterogeneous data resources [33–36], it is still challenging to balance coverage against quality in network integration. Consequently, sparking new ideas in data digging and integration is crucial to make a breakthrough in disease gene discovery. Also, we would like to see in the future that most data sources can annotate genes with standardized and objective vocabularies like GO database does, which will definitely facilitate data interoperation and fusion. At last, innovation of computational tools is in desperate need. Current algorithms mainly rely on RWR to globally infer topological distance, whereas this method is well biased towards hub nodes [26]. Future work should pay more attention to alleviate this kind of bias.

## Additional files

Additional file 1: Table S1 The 633 disease genes corresponding to the 113 phenotypes collected from the OMIM database. **Table S2.** The 572 disease genes corresponding to the 54 diseases which are included in the DisGeNET database (DOCX 68 kb)
