# Supplementary material for: Gene gravity-like algorithm for disease gene prediction based on phenotype-specific network
Source: BMC Syst Biol. 2017 Dec 6;11:121. doi: 10.1186/s12918-017-0519-9 (PMC5718078; doi:10.1186/s12918-017-0519-9)
Supplement: Supplementary file 1 — The 633 disease genes corresponding to the 113 phenotypes collected from the OMIM database. Table S2. The 572 disease genes corresponding to the 54 diseases which are included in the DisGeNET database (DOCX 68 kb) [file 12918_2017_519_MOESM1_ESM.docx]

# Additional file for “Gene gravity-like algorithm for disease gene prediction based on phenotype-specific network”

**Contents**

Table S1. The 633 disease genes corresponding to the 113 phenotypes collected from the OMIM database.

Table S2. The 572 disease genes corresponding to the 54 diseases which are included in the DisGeNET database.

Table S1. The 633 disease genes corresponding to the 113 phenotypes collected from the OMIM database.

| OMIM ID | Phenotype | Gene symbol | Entrez ID |
| --- | --- | --- | --- |
| 125853 | Diabetes mellitus | AKT2 | 208 |
| 125853 | Diabetes mellitus | GCGR | 2642 |
| 125853 | Diabetes mellitus | GCK | 2645 |
| 125853 | Diabetes mellitus | GPD2 | 2820 |
| 125853 | Diabetes mellitus | HMGA1 | 3159 |
| 125853 | Diabetes mellitus | HNF4A | 3172 |
| 125853 | Diabetes mellitus | IL6 | 3569 |
| 125853 | Diabetes mellitus | PDX1 | 3651 |
| 125853 | Diabetes mellitus | IRS1 | 3667 |
| 125853 | Diabetes mellitus | KCNJ11 | 3767 |
| 125853 | Diabetes mellitus | LIPC | 3990 |
| 125853 | Diabetes mellitus | MTNR1B | 4544 |
| 125853 | Diabetes mellitus | NEUROD1 | 4760 |
| 125853 | Diabetes mellitus | PAX4 | 5078 |
| 125853 | Diabetes mellitus | ENPP1 | 5167 |
| 125853 | Diabetes mellitus | PPARG | 5468 |
| 125853 | Diabetes mellitus | PTPN1 | 5770 |
| 125853 | Diabetes mellitus | SLC2A2 | 6514 |
| 125853 | Diabetes mellitus | ABCC8 | 6833 |
| 125853 | Diabetes mellitus | HNF1A | 6927 |
| 125853 | Diabetes mellitus | HNF1B | 6928 |
| 125853 | Diabetes mellitus | TCF7L2 | 6934 |
| 125853 | Diabetes mellitus | WFS1 | 7466 |
| 125853 | Diabetes mellitus | IRS2 | 8660 |
| 125853 | Diabetes mellitus | MAPK8IP1 | 9479 |
| 125853 | Diabetes mellitus | IGF2BP2 | 10644 |
| 125853 | Diabetes mellitus | CDKAL1 | 54901 |
| 125853 | Diabetes mellitus | RETN | 56729 |
| 125853 | Diabetes mellitus | SLC30A8 | 169026 |
| 114500 | Colon cancer | AKT1 | 207 |
| 114500 | Colon cancer | APC | 324 |
| 114500 | Colon cancer | BAX | 581 |
| 114500 | Colon cancer | CCND1 | 595 |
| 114500 | Colon cancer | BUB1B | 701 |
| 114500 | Colon cancer | CTNNB1 | 1499 |
| 114500 | Colon cancer | DCC | 1630 |
| 114500 | Colon cancer | EP300 | 2033 |
| 114500 | Colon cancer | FGFR3 | 2261 |
| 114500 | Colon cancer | MCC | 4163 |
| 114500 | Colon cancer | NRAS | 4893 |
| 114500 | Colon cancer | ODC1 | 4953 |
| 114500 | Colon cancer | PDGFRL | 5157 |
| 114500 | Colon cancer | PIK3CA | 5290 |
| 114500 | Colon cancer | PLA2G2A | 5320 |
| 114500 | Colon cancer | PTPN12 | 5782 |
| 114500 | Colon cancer | PTPRJ | 5795 |
| 114500 | Colon cancer | AURKA | 6790 |
| 114500 | Colon cancer | TLR2 | 7097 |
| 114500 | Colon cancer | TLR4 | 7099 |
| 114500 | Colon cancer | TP53 | 7157 |
| 114500 | Colon cancer | AXIN2 | 8313 |
| 114500 | Colon cancer | DLC1 | 10395 |
| 114500 | Colon cancer | RAD54B | 25788 |
| 114500 | Colon cancer | MLH3 | 27030 |
| 114500 | Colon cancer | FLCN | 201163 |
| 114480 | Breast cancer | AKT1 | 207 |
| 114480 | Breast cancer | ATM | 472 |
| 114480 | Breast cancer | BARD1 | 580 |
| 114480 | Breast cancer | BRCA2 | 675 |
| 114480 | Breast cancer | CASP8 | 841 |
| 114480 | Breast cancer | CDH1 | 999 |
| 114480 | Breast cancer | ESR1 | 2099 |
| 114480 | Breast cancer | HMMR | 3161 |
| 114480 | Breast cancer | KRAS | 3845 |
| 114480 | Breast cancer | NQO2 | 4835 |
| 114480 | Breast cancer | SLC22A18 | 5002 |
| 114480 | Breast cancer | PHB | 5245 |
| 114480 | Breast cancer | PIK3CA | 5290 |
| 114480 | Breast cancer | RAD51 | 5888 |
| 114480 | Breast cancer | TP53 | 7157 |
| 114480 | Breast cancer | TSG101 | 7251 |
| 114480 | Breast cancer | XRCC3 | 7517 |
| 114480 | Breast cancer | RAD54L | 8438 |
| 114480 | Breast cancer | PPM1D | 8493 |
| 114480 | Breast cancer | RB1CC1 | 9821 |
| 114480 | Breast cancer | CHEK2 | 11200 |
| 114480 | Breast cancer | BRIP1 | 83990 |
| 601626 | Leukemia | RUNX1 | 861 |
| 601626 | Leukemia | CBFB | 865 |
| 601626 | Leukemia | CEBPA | 1050 |
| 601626 | Leukemia | ETV6 | 2120 |
| 601626 | Leukemia | FLT3 | 2322 |
| 601626 | Leukemia | GATA2 | 2624 |
| 601626 | Leukemia | JAK2 | 3717 |
| 601626 | Leukemia | KIT | 3815 |
| 601626 | Leukemia | KRAS | 3845 |
| 601626 | Leukemia | LPP | 4026 |
| 601626 | Leukemia | MLF1 | 4291 |
| 601626 | Leukemia | NPM1 | 4869 |
| 601626 | Leukemia | SH3GL1 | 6455 |
| 601626 | Leukemia | TERT | 7015 |
| 601626 | Leukemia | NUP214 | 8021 |
| 601626 | Leukemia | MLLT10 | 8028 |
| 601626 | Leukemia | PICALM | 8301 |
| 601626 | Leukemia | CHIC2 | 26511 |
| 601626 | Leukemia | WHSC1L1 | 54904 |
| 601626 | Leukemia | NSD1 | 64324 |
| 252010 | Mitochondrial complex I deficiency | NDUFA1 | 4694 |
| 252010 | Mitochondrial complex I deficiency | NDUFB3 | 4709 |
| 252010 | Mitochondrial complex I deficiency | NDUFB9 | 4715 |
| 252010 | Mitochondrial complex I deficiency | NDUFS1 | 4719 |
| 252010 | Mitochondrial complex I deficiency | NDUFS2 | 4720 |
| 252010 | Mitochondrial complex I deficiency | NDUFS3 | 4722 |
| 252010 | Mitochondrial complex I deficiency | NDUFV1 | 4723 |
| 252010 | Mitochondrial complex I deficiency | NDUFS4 | 4724 |
| 252010 | Mitochondrial complex I deficiency | NDUFS6 | 4726 |
| 252010 | Mitochondrial complex I deficiency | NDUFV2 | 4729 |
| 252010 | Mitochondrial complex I deficiency | NDUFAF3 | 25915 |
| 252010 | Mitochondrial complex I deficiency | NDUFAF4 | 29078 |
| 252010 | Mitochondrial complex I deficiency | NDUFAF1 | 51103 |
| 252010 | Mitochondrial complex I deficiency | FOXRED1 | 55572 |
| 252010 | Mitochondrial complex I deficiency | NDUFAF5 | 79133 |
| 252010 | Mitochondrial complex I deficiency | NUBPL | 80224 |
| 252010 | Mitochondrial complex I deficiency | NDUFAF2 | 91942 |
| 252010 | Mitochondrial complex I deficiency | NDUFA11 | 126328 |
| 211980 | Lung cancer | FASLG | 356 |
| 211980 | Lung cancer | BRAF | 673 |
| 211980 | Lung cancer | CASP8 | 841 |
| 211980 | Lung cancer | MAP3K8 | 1326 |
| 211980 | Lung cancer | CYP2A6 | 1548 |
| 211980 | Lung cancer | EGFR | 1956 |
| 211980 | Lung cancer | ERBB2 | 2064 |
| 211980 | Lung cancer | ERCC6 | 2074 |
| 211980 | Lung cancer | IRF1 | 3659 |
| 211980 | Lung cancer | KRAS | 3845 |
| 211980 | Lung cancer | SLC22A18 | 5002 |
| 211980 | Lung cancer | PARK2 | 5071 |
| 211980 | Lung cancer | PIK3CA | 5290 |
| 211980 | Lung cancer | PPP2R1B | 5519 |
| 211980 | Lung cancer | DLEC1 | 9940 |
| 211980 | Lung cancer | RASSF1 | 11186 |
| 256000 | Leigh syndrome | BCS1L | 617 |
| 256000 | Leigh syndrome | COX10 | 1352 |
| 256000 | Leigh syndrome | COX15 | 1355 |
| 256000 | Leigh syndrome | NDUFA2 | 4695 |
| 256000 | Leigh syndrome | NDUFA9 | 4704 |
| 256000 | Leigh syndrome | NDUFA10 | 4705 |
| 256000 | Leigh syndrome | NDUFS3 | 4722 |
| 256000 | Leigh syndrome | NDUFS4 | 4724 |
| 256000 | Leigh syndrome | NDUFS8 | 4728 |
| 256000 | Leigh syndrome | SDHA | 6389 |
| 256000 | Leigh syndrome | SURF1 | 6834 |
| 256000 | Leigh syndrome | FOXRED1 | 55572 |
| 256000 | Leigh syndrome | NDUFA12 | 55967 |
| 256000 | Leigh syndrome | NDUFAF2 | 91942 |
| 256000 | Leigh syndrome | NDUFAF6 | 137682 |
| 256000 | Leigh syndrome | NDUFS7 | 374291 |
| 181500 | Schizophrenia | AKT1 | 207 |
| 181500 | Schizophrenia | CHI3L1 | 1116 |
| 181500 | Schizophrenia | COMT | 1312 |
| 181500 | Schizophrenia | DAO | 1610 |
| 181500 | Schizophrenia | DRD3 | 1814 |
| 181500 | Schizophrenia | HTR2A | 3356 |
| 181500 | Schizophrenia | MTHFR | 4524 |
| 181500 | Schizophrenia | SYN2 | 6854 |
| 181500 | Schizophrenia | APOL2 | 23780 |
| 181500 | Schizophrenia | DISC1 | 27185 |
| 181500 | Schizophrenia | RTN4R | 65078 |
| 181500 | Schizophrenia | DTNBP1 | 84062 |
| 601665 | Obesity | ADRB2 | 154 |
| 601665 | Obesity | ADRB3 | 155 |
| 601665 | Obesity | AGRP | 181 |
| 601665 | Obesity | MC4R | 4160 |
| 601665 | Obesity | ENPP1 | 5167 |
| 601665 | Obesity | POMC | 5443 |
| 601665 | Obesity | PPARG | 5468 |
| 601665 | Obesity | SIM1 | 6492 |
| 601665 | Obesity | UCP1 | 7350 |
| 601665 | Obesity | UCP3 | 7352 |
| 601665 | Obesity | NR0B2 | 8431 |
| 601665 | Obesity | CARTPT | 9607 |
| 601665 | Obesity | SDC3 | 9672 |
| 601665 | Obesity | GHRL | 51738 |
| 601665 | Obesity | PPARGC1B | 133522 |
| 608446 | Myocardial infarcation | ESR1 | 2099 |
| 608446 | Myocardial infarcation | F7 | 2155 |
| 608446 | Myocardial infarcation | F13A1 | 2162 |
| 608446 | Myocardial infarcation | GCLC | 2729 |
| 608446 | Myocardial infarcation | GCLM | 2730 |
| 608446 | Myocardial infarcation | ITGB3 | 3690 |
| 608446 | Myocardial infarcation | LGALS2 | 3957 |
| 608446 | Myocardial infarcation | LTA | 4049 |
| 608446 | Myocardial infarcation | OLR1 | 4973 |
| 608446 | Myocardial infarcation | PSMA6 | 5687 |
| 608446 | Myocardial infarcation | TNFSF4 | 7292 |
| 608446 | Myocardial infarcation | LRP8 | 7804 |
| 145500 | Hypertension | ADD1 | 118 |
| 145500 | Hypertension | AGT | 183 |
| 145500 | Hypertension | AGTR1 | 185 |
| 145500 | Hypertension | ATP1B1 | 481 |
| 145500 | Hypertension | CYP3A5 | 1577 |
| 145500 | Hypertension | ECE1 | 1889 |
| 145500 | Hypertension | GNB3 | 2784 |
| 145500 | Hypertension | NOS2 | 4843 |
| 145500 | Hypertension | NOS3 | 4846 |
| 145500 | Hypertension | PTGIS | 5740 |
| 145500 | Hypertension | SELE | 6401 |
| 145500 | Hypertension | RGS5 | 8490 |
| 176807 | Prostate cancer | AR | 367 |
| 176807 | Prostate cancer | ZFHX3 | 463 |
| 176807 | Prostate cancer | BRCA2 | 675 |
| 176807 | Prostate cancer | CDH1 | 999 |
| 176807 | Prostate cancer | KLF6 | 1316 |
| 176807 | Prostate cancer | HIP1 | 3092 |
| 176807 | Prostate cancer | CD82 | 3732 |
| 176807 | Prostate cancer | MSR1 | 4481 |
| 176807 | Prostate cancer | MXI1 | 4601 |
| 176807 | Prostate cancer | PTEN | 5728 |
| 176807 | Prostate cancer | MAD1L1 | 8379 |
| 176807 | Prostate cancer | CHEK2 | 11200 |
| 104300 | Alzheimer disease | A2M | 2 |
| 104300 | Alzheimer disease | APBB2 | 323 |
| 104300 | Alzheimer disease | APP | 351 |
| 104300 | Alzheimer disease | BLMH | 642 |
| 104300 | Alzheimer disease | ACE | 1636 |
| 104300 | Alzheimer disease | HFE | 3077 |
| 104300 | Alzheimer disease | MPO | 4353 |
| 104300 | Alzheimer disease | NOS3 | 4846 |
| 104300 | Alzheimer disease | PLAU | 5328 |
| 104300 | Alzheimer disease | SORL1 | 6653 |
| 104300 | Alzheimer disease | PAXIP1 | 22976 |
| 600807 | Asthma | ADRB2 | 154 |
| 600807 | Asthma | ALOX5 | 240 |
| 600807 | Asthma | HLA-G | 3135 |
| 600807 | Asthma | HNMT | 3176 |
| 600807 | Asthma | IL13 | 3596 |
| 600807 | Asthma | MUC7 | 4589 |
| 600807 | Asthma | CCL11 | 6356 |
| 600807 | Asthma | TNF | 7124 |
| 600807 | Asthma | PLA2G7 | 7941 |
| 600807 | Asthma | PHF11 | 51131 |
| 600807 | Asthma | SCGB3A2 | 117156 |
| 607948 | Mycobacterium tuberculosis | CISH | 1154 |
| 607948 | Mycobacterium tuberculosis | SP110 | 3431 |
| 607948 | Mycobacterium tuberculosis | IFNG | 3458 |
| 607948 | Mycobacterium tuberculosis | IFNGR1 | 3459 |
| 607948 | Mycobacterium tuberculosis | MC3R | 4159 |
| 607948 | Mycobacterium tuberculosis | CCL2 | 6347 |
| 607948 | Mycobacterium tuberculosis | SLC11A1 | 6556 |
| 607948 | Mycobacterium tuberculosis | TLR2 | 7097 |
| 607948 | Mycobacterium tuberculosis | CD209 | 30835 |
| 607948 | Mycobacterium tuberculosis | TIRAP | 114609 |
| 220110 | Mitochondrial complex IV deficiency | COX6B1 | 1340 |
| 220110 | Mitochondrial complex IV deficiency | COX8A | 1351 |
| 220110 | Mitochondrial complex IV deficiency | COX10 | 1352 |
| 220110 | Mitochondrial complex IV deficiency | SCO1 | 6341 |
| 220110 | Mitochondrial complex IV deficiency | FASTKD2 | 22868 |
| 220110 | Mitochondrial complex IV deficiency | TACO1 | 51204 |
| 220110 | Mitochondrial complex IV deficiency | APOPT1 | 84334 |
| 220110 | Mitochondrial complex IV deficiency | COX20 | 116228 |
| 114550 | Hepatoblastoma | APC | 324 |
| 114550 | Hepatoblastoma | CASP8 | 841 |
| 114550 | Hepatoblastoma | CTNNB1 | 1499 |
| 114550 | Hepatoblastoma | IGF2R | 3482 |
| 114550 | Hepatoblastoma | MET | 4233 |
| 114550 | Hepatoblastoma | PDGFRL | 5157 |
| 114550 | Hepatoblastoma | PIK3CA | 5290 |
| 114550 | Hepatoblastoma | TP53 | 7157 |
| 114550 | Hepatoblastoma | AXIN1 | 8312 |
| 147050 | Atopy | MS4A2 | 2206 |
| 147050 | Atopy | IL4R | 3566 |
| 147050 | Atopy | SELP | 6403 |
| 147050 | Atopy | PLA2G7 | 7941 |
| 147050 | Atopy | SPINK5 | 11005 |
| 147050 | Atopy | HAVCR1 | 26762 |
| 147050 | Atopy | IL21R | 50615 |
| 147050 | Atopy | PHF11 | 51131 |
| 171300 | Pheochromocytoma | GDNF | 2668 |
| 171300 | Pheochromocytoma | MAX | 4149 |
| 171300 | Pheochromocytoma | RET | 5979 |
| 171300 | Pheochromocytoma | SDHB | 6390 |
| 171300 | Pheochromocytoma | SDHD | 6392 |
| 171300 | Pheochromocytoma | VHL | 7428 |
| 171300 | Pheochromocytoma | KIF1B | 23095 |
| 171300 | Pheochromocytoma | TMEM127 | 55654 |
| 180300 | Rheumatoid arthritis | HLA-DRB1 | 3123 |
| 180300 | Rheumatoid arthritis | IL10 | 3586 |
| 180300 | Rheumatoid arthritis | CIITA | 4261 |
| 180300 | Rheumatoid arthritis | NFKBIL1 | 4795 |
| 180300 | Rheumatoid arthritis | SLC22A4 | 6583 |
| 180300 | Rheumatoid arthritis | PADI4 | 23569 |
| 180300 | Rheumatoid arthritis | PTPN22 | 26191 |
| 180300 | Rheumatoid arthritis | CD244 | 51744 |
| 133239 | Esophageal cancer | DCC | 1630 |
| 133239 | Esophageal cancer | RNF6 | 6049 |
| 133239 | Esophageal cancer | TGFBR2 | 7048 |
| 133239 | Esophageal cancer | DLEC1 | 9940 |
| 133239 | Esophageal cancer | LZTS1 | 11178 |
| 133239 | Esophageal cancer | 1-Dec | 50514 |
| 133239 | Esophageal cancer | WWOX | 51741 |
| 143890 | Hypercholesterolemia | ABCA1 | 19 |
| 143890 | Hypercholesterolemia | APOA2 | 336 |
| 143890 | Hypercholesterolemia | EPHX2 | 2053 |
| 143890 | Hypercholesterolemia | GHR | 2690 |
| 143890 | Hypercholesterolemia | ITIH4 | 3700 |
| 143890 | Hypercholesterolemia | LDLR | 3949 |
| 143890 | Hypercholesterolemia | PPP1R17 | 10842 |
| 144700 | Renal cell carcinoma | OGG1 | 4968 |
| 144700 | Renal cell carcinoma | HNF1A | 6927 |
| 144700 | Renal cell carcinoma | HNF1B | 6928 |
| 144700 | Renal cell carcinoma | VHL | 7428 |
| 144700 | Renal cell carcinoma | RNF139 | 11236 |
| 144700 | Renal cell carcinoma | DIRC2 | 84925 |
| 144700 | Renal cell carcinoma | FLCN | 201163 |
| 187500 | Tetralogy of Fallot | JAG1 | 182 |
| 187500 | Tetralogy of Fallot | NKX2-5 | 1482 |
| 187500 | Tetralogy of Fallot | GATA4 | 2626 |
| 187500 | Tetralogy of Fallot | GATA6 | 2627 |
| 187500 | Tetralogy of Fallot | GDF1 | 2657 |
| 187500 | Tetralogy of Fallot | TBX1 | 6899 |
| 187500 | Tetralogy of Fallot | ZFPM2 | 23414 |
| 103780 | Alcohol dependence | ADH1B | 125 |
| 103780 | Alcohol dependence | ADH1C | 126 |
| 103780 | Alcohol dependence | GABRA2 | 2555 |
| 103780 | Alcohol dependence | HTR2A | 3356 |
| 103780 | Alcohol dependence | RCBTB1 | 55213 |
| 152700 | Lupus nephritis | CTLA4 | 1493 |
| 152700 | Lupus nephritis | DNASE1 | 1773 |
| 152700 | Lupus nephritis | FCGR2A | 2212 |
| 152700 | Lupus nephritis | FCGR2B | 2213 |
| 152700 | Lupus nephritis | TREX1 | 11277 |
| 152700 | Lupus nephritis | PTPN22 | 26191 |
| 166710 | Osteoporosis | CALCR | 799 |
| 166710 | Osteoporosis | COL1A1 | 1277 |
| 166710 | Osteoporosis | COL1A2 | 1278 |
| 166710 | Osteoporosis | LRP5 | 4041 |
| 166710 | Osteoporosis | VDR | 7421 |
| 166710 | Osteoporosis | PDLIM4 | 8572 |
| 209880 | Central hypoventilation syndrome | ASCL1 | 429 |
| 209880 | Central hypoventilation syndrome | BDNF | 627 |
| 209880 | Central hypoventilation syndrome | EDN3 | 1908 |
| 209880 | Central hypoventilation syndrome | GDNF | 2668 |
| 209880 | Central hypoventilation syndrome | RET | 5979 |
| 209880 | Central hypoventilation syndrome | PHOX2B | 8929 |
| 603932 | Intervertebral disc disease | COL9A2 | 1298 |
| 603932 | Intervertebral disc disease | COL9A3 | 1299 |
| 603932 | Intervertebral disc disease | COL11A1 | 1301 |
| 603932 | Intervertebral disc disease | THBS2 | 7058 |
| 603932 | Intervertebral disc disease | CILP | 8483 |
| 603932 | Intervertebral disc disease | ASPN | 54829 |
| 607174 | Meningioma | MN1 | 4330 |
| 607174 | Meningioma | NF2 | 4771 |
| 607174 | Meningioma | PDGFB | 5155 |
| 607174 | Meningioma | PTEN | 5728 |
| 607174 | Meningioma | SMARCE1 | 6605 |
| 607174 | Meningioma | SUFU | 51684 |
| 167800 | Pancreatitis | CFTR | 1080 |
| 167800 | Pancreatitis | PRSS1 | 5644 |
| 167800 | Pancreatitis | PRSS2 | 5645 |
| 167800 | Pancreatitis | SPINK1 | 6690 |
| 167800 | Pancreatitis | CTRC | 11330 |
| 168600 | Parkinson disease | ADH1C | 126 |
| 168600 | Parkinson disease | GBA | 2629 |
| 168600 | Parkinson disease | MAPT | 4137 |
| 168600 | Parkinson disease | ATXN2 | 6311 |
| 168600 | Parkinson disease | TBP | 6908 |
| 182940 | Neural tube defects | CCL2 | 6347 |
| 182940 | Neural tube defects | T | 6862 |
| 182940 | Neural tube defects | VANGL2 | 57216 |
| 182940 | Neural tube defects | FUZ | 80199 |
| 182940 | Neural tube defects | VANGL1 | 81839 |
| 217095 | Conotruncal anomaly face syndrome | NKX2-5 | 1482 |
| 217095 | Conotruncal anomaly face syndrome | GATA6 | 2627 |
| 217095 | Conotruncal anomaly face syndrome | GDF1 | 2657 |
| 217095 | Conotruncal anomaly face syndrome | TBX1 | 6899 |
| 222100 | Diabetes, type 1 | ITPR3 | 3710 |
| 222100 | Diabetes, type 1 | OAS1 | 4938 |
| 222100 | Diabetes, type 1 | HNF1A | 6927 |
| 222100 | Diabetes, type 1 | PTPN22 | 26191 |
| 222100 | Diabetes, type 1 | FOXP3 | 50943 |
| 226650 | Epidermolysis bullosa | COL17A1 | 1308 |
| 226650 | Epidermolysis bullosa | ITGB4 | 3691 |
| 226650 | Epidermolysis bullosa | LAMA3 | 3909 |
| 226650 | Epidermolysis bullosa | LAMB3 | 3914 |
| 226650 | Epidermolysis bullosa | LAMC2 | 3918 |
| 601367 | Cerebral infarction | ALOX5AP | 241 |
| 601367 | Cerebral infarction | F2 | 2147 |
| 601367 | Cerebral infarction | F5 | 2153 |
| 601367 | Cerebral infarction | NOS3 | 4846 |
| 601367 | Cerebral infarction | PRKCH | 5583 |
| 603896 | Leukoencephalopathy with vanishing white matter | EIF2B1 | 1967 |
| 603896 | Leukoencephalopathy with vanishing white matter | EIF2B4 | 8890 |
| 603896 | Leukoencephalopathy with vanishing white matter | EIF2B3 | 8891 |
| 603896 | Leukoencephalopathy with vanishing white matter | EIF2B2 | 8892 |
| 603896 | Leukoencephalopathy with vanishing white matter | EIF2B5 | 8893 |
| 605027 | Lymphoma, follicular | CASP10 | 843 |
| 605027 | Lymphoma, follicular | PRF1 | 5551 |
| 605027 | Lymphoma, follicular | RAD54L | 8438 |
| 605027 | Lymphoma, follicular | BCL10 | 8915 |
| 605027 | Lymphoma, follicular | RAD54B | 25788 |
| 105200 | Amyloidosis, familial visceral | APOA1 | 335 |
| 105200 | Amyloidosis, familial visceral | B2M | 567 |
| 105200 | Amyloidosis, familial visceral | FGA | 2243 |
| 105200 | Amyloidosis, familial visceral | LYZ | 4069 |
| 105400 | Amyotrophic lateral sclerosis | DCTN1 | 1639 |
| 105400 | Amyotrophic lateral sclerosis | NEFH | 4744 |
| 105400 | Amyotrophic lateral sclerosis | PRPH | 5630 |
| 105400 | Amyotrophic lateral sclerosis | SOD1 | 6647 |
| 109800 | Bladder cancer | FGFR3 | 2261 |
| 109800 | Bladder cancer | HRAS | 3265 |
| 109800 | Bladder cancer | KRAS | 3845 |
| 109800 | Bladder cancer | RB1 | 5925 |
| 122700 | Warfarin sensitivity | CYP2A6 | 1548 |
| 122700 | Warfarin sensitivity | CYP2C9 | 1559 |
| 122700 | Warfarin sensitivity | F9 | 2158 |
| 122700 | Warfarin sensitivity | VKORC1 | 79001 |
| 136880 | Fundus albipunctatus | RDH5 | 5959 |
| 136880 | Fundus albipunctatus | PRPH2 | 5961 |
| 136880 | Fundus albipunctatus | RHO | 6010 |
| 136880 | Fundus albipunctatus | RLBP1 | 6017 |
| 137215 | Gastric cancer | CDH1 | 999 |
| 137215 | Gastric cancer | IL1B | 3553 |
| 137215 | Gastric cancer | IL1RN | 3557 |
| 137215 | Gastric cancer | KRAS | 3845 |
| 145900 | Dejerine-Sottas disease | EGR2 | 1959 |
| 145900 | Dejerine-Sottas disease | MPZ | 4359 |
| 145900 | Dejerine-Sottas disease | PMP22 | 5376 |
| 145900 | Dejerine-Sottas disease | PRX | 57716 |
| 162900 | Dejerine-Sottas disease | FGFR3 | 2261 |
| 162900 | Dejerine-Sottas disease | HRAS | 3265 |
| 162900 | Dejerine-Sottas disease | NRAS | 4893 |
| 162900 | Dejerine-Sottas disease | PIK3CA | 5290 |
| 188050 | Thromboembolism | F2 | 2147 |
| 188050 | Thromboembolism | F13A1 | 2162 |
| 188050 | Thromboembolism | HABP2 | 3026 |
| 188050 | Thromboembolism | MTHFR | 4524 |
| 188470 | Thyroid cancer | HRAS | 3265 |
| 188470 | Thyroid cancer | NRAS | 4893 |
| 188470 | Thyroid cancer | MINPP1 | 9562 |
| 188470 | Thyroid cancer | SRGAP1 | 57522 |
| 188890 | Nicotine addiction | CHRNA4 | 1137 |
| 188890 | Nicotine addiction | CYP2A6 | 1548 |
| 188890 | Nicotine addiction | SLC6A3 | 6531 |
| 188890 | Nicotine addiction | GABBR2 | 9568 |
| 209920 | Bare lymphocyte syndrome | CIITA | 4261 |
| 209920 | Bare lymphocyte syndrome | RFX5 | 5993 |
| 209920 | Bare lymphocyte syndrome | RFXAP | 5994 |
| 209920 | Bare lymphocyte syndrome | RFXANK | 8625 |
| 254450 | Myelofibrosis | CALR | 811 |
| 254450 | Myelofibrosis | JAK2 | 3717 |
| 254450 | Myelofibrosis | MPL | 4352 |
| 254450 | Myelofibrosis | SH2B3 | 10019 |
| 260350 | Pancreatic cancer | KRAS | 3845 |
| 260350 | Pancreatic cancer | SMAD4 | 4089 |
| 260350 | Pancreatic cancer | STK11 | 6794 |
| 260350 | Pancreatic cancer | TP53 | 7157 |
| 267430 | Renal tubular dysgenesis | AGT | 183 |
| 267430 | Renal tubular dysgenesis | AGTR1 | 185 |
| 267430 | Renal tubular dysgenesis | ACE | 1636 |
| 267430 | Renal tubular dysgenesis | REN | 5972 |
| 276300 | Mismatch repair cancer syndrome | MSH6 | 2956 |
| 276300 | Mismatch repair cancer syndrome | MLH1 | 4292 |
| 276300 | Mismatch repair cancer syndrome | MSH2 | 4436 |
| 276300 | Mismatch repair cancer syndrome | PMS2 | 5395 |
| 601634 | Neural tube defects | MTHFD1 | 4522 |
| 601634 | Neural tube defects | MTHFR | 4524 |
| 601634 | Neural tube defects | MTR | 4548 |
| 601634 | Neural tube defects | MTRR | 4552 |
| 601680 | Arthrogryposis multiplex congenita | MYH3 | 4621 |
| 601680 | Arthrogryposis multiplex congenita | TNNI2 | 7136 |
| 601680 | Arthrogryposis multiplex congenita | TNNT3 | 7140 |
| 601680 | Arthrogryposis multiplex congenita | TPM2 | 7169 |
| 606176 | Diabetes mellitus | GCK | 2645 |
| 606176 | Diabetes mellitus | INS | 3630 |
| 606176 | Diabetes mellitus | KCNJ11 | 3767 |
| 606176 | Diabetes mellitus | ABCC8 | 6833 |
| 606764 | Gastrointestinal stromal tumor | KIT | 3815 |
| 606764 | Gastrointestinal stromal tumor | PDGFRA | 5156 |
| 606764 | Gastrointestinal stromal tumor | SDHB | 6390 |
| 606764 | Gastrointestinal stromal tumor | SDHC | 6391 |
| 607785 | Leukemia | NF1 | 4763 |
| 607785 | Leukemia | PTPN11 | 5781 |
| 607785 | Leukemia | MLLT11 | 10962 |
| 607785 | Leukemia | ARHGAP26 | 23092 |
| 109400 | Basal cell nevus syndrome | PTCH1 | 5727 |
| 109400 | Basal cell nevus syndrome | PTCH2 | 8643 |
| 109400 | Basal cell nevus syndrome | SUFU | 51684 |
| 112600 | Brachydactyly | BMP2 | 650 |
| 112600 | Brachydactyly | BMPR1B | 658 |
| 112600 | Brachydactyly | GDF5 | 8200 |
| 126200 | Multiple sclerosis | HLA-DQB1 | 3119 |
| 126200 | Multiple sclerosis | HLA-DRB1 | 3123 |
| 126200 | Multiple sclerosis | PDCD1 | 5133 |
| 127750 | Lewy body dementia | GBA | 2629 |
| 127750 | Lewy body dementia | SNCB | 6620 |
| 127750 | Lewy body dementia | SNCA | 6622 |
| 130000 | Ehlers-Danlos syndrome | COL1A1 | 1277 |
| 130000 | Ehlers-Danlos syndrome | COL5A1 | 1289 |
| 130000 | Ehlers-Danlos syndrome | COL5A2 | 1290 |
| 131800 | Epidermolysis bullosa of hands and feet | ITGB4 | 3691 |
| 131800 | Epidermolysis bullosa of hands and feet | KRT5 | 3852 |
| 131800 | Epidermolysis bullosa of hands and feet | KRT14 | 3861 |
| 133100 | [Erythrocytosis, familial | EPOR | 2057 |
| 133100 | [Erythrocytosis, familial | JAK2 | 3717 |
| 133100 | [Erythrocytosis, familial | SH2B3 | 10019 |
| 133200 | Erythrokeratodermia variabilis et progressiva | GJA1 | 2697 |
| 133200 | Erythrokeratodermia variabilis et progressiva | GJB3 | 2707 |
| 133200 | Erythrokeratodermia variabilis et progressiva | GJB4 | 127534 |
| 137800 | Glioma susceptibility 1 | ERBB2 | 2064 |
| 137800 | Glioma susceptibility 1 | IDH1 | 3417 |
| 137800 | Glioma susceptibility 1 | TP53 | 7157 |
| 138500 | Hyperglycinuria | SLC6A20 | 54716 |
| 138500 | Hyperglycinuria | SLC36A2 | 153201 |
| 138500 | Hyperglycinuria | SLC6A19 | 340024 |
| 140700 | Heinz body anemia | HBA1 | 3039 |
| 140700 | Heinz body anemia | HBA2 | 3040 |
| 140700 | Heinz body anemia | HBB | 3043 |
| 141749 | Fetal hemoglobin quantitative trait locus 1 | HBB | 3043 |
| 141749 | Fetal hemoglobin quantitative trait locus 1 | HBG1 | 3047 |
| 141749 | Fetal hemoglobin quantitative trait locus 1 | HBG2 | 3048 |
| 149730 | LADD syndrome | FGF10 | 2255 |
| 149730 | LADD syndrome | FGFR3 | 2261 |
| 149730 | LADD syndrome | FGFR2 | 2263 |
| 155255 | Medulloblastoma | BRCA2 | 675 |
| 155255 | Medulloblastoma | PTCH2 | 8643 |
| 155255 | Medulloblastoma | SUFU | 51684 |
| 157300 | Migraine without aura | EDNRA | 1909 |
| 157300 | Migraine without aura | ESR1 | 2099 |
| 157300 | Migraine without aura | TNF | 7124 |
| 158000 | Monilethrix | KRT81 | 3887 |
| 158000 | Monilethrix | KRT83 | 3889 |
| 158000 | Monilethrix | KRT86 | 3892 |
| 158810 | Bethlem myopathy 1 | COL6A1 | 1291 |
| 158810 | Bethlem myopathy 1 | COL6A2 | 1292 |
| 158810 | Bethlem myopathy 1 | COL6A3 | 1293 |
| 163200 | Schimmelpenning-Feuerstein-Mims syndrome | HRAS | 3265 |
| 163200 | Schimmelpenning-Feuerstein-Mims syndrome | KRAS | 3845 |
| 163200 | Schimmelpenning-Feuerstein-Mims syndrome | NRAS | 4893 |
| 164230 | Obsessive-compulsive disorder | BDNF | 627 |
| 164230 | Obsessive-compulsive disorder | HTR2A | 3356 |
| 164230 | Obsessive-compulsive disorder | SLC6A4 | 6532 |
| 187950 | Thrombocythemia 1 | CALR | 811 |
| 187950 | Thrombocythemia 1 | THPO | 7066 |
| 187950 | Thrombocythemia 1 | SH2B3 | 10019 |
| 192600 | Cardiomyopathy | CAV3 | 859 |
| 192600 | Cardiomyopathy | MYH7 | 4625 |
| 192600 | Cardiomyopathy | MYLK2 | 85366 |
| 194070 | Wilms tumor | BRCA2 | 675 |
| 194070 | Wilms tumor | GPC3 | 2719 |
| 194070 | Wilms tumor | WT1 | 7490 |
| 202400 | Afibrinogenemia | FGA | 2243 |
| 202400 | Afibrinogenemia | FGB | 2244 |
| 202400 | Afibrinogenemia | FGG | 2266 |
| 211900 | Tumoral calcinosis | GALNT3 | 2591 |
| 211900 | Tumoral calcinosis | FGF23 | 8074 |
| 211900 | Tumoral calcinosis | KL | 9365 |
| 216360 | COACH syndrome | RPGRIP1L | 23322 |
| 216360 | COACH syndrome | CC2D2A | 57545 |
| 216360 | COACH syndrome | TMEM67 | 91147 |
| 219700 | Cystic fibrosis lung disease | CFTR | 1080 |
| 219700 | Cystic fibrosis lung disease | FCGR2A | 2212 |
| 219700 | Cystic fibrosis lung disease | TGFB1 | 7040 |
| 220290 | Deafness | GJB2 | 2706 |
| 220290 | Deafness | GJB3 | 2707 |
| 220290 | Deafness | GJB6 | 10804 |
| 226700 | Epidermolysis bullosa | LAMA3 | 3909 |
| 226700 | Epidermolysis bullosa | LAMB3 | 3914 |
| 226700 | Epidermolysis bullosa | LAMC2 | 3918 |
| 231200 | Bernard-Soulier syndrome | GP1BA | 2811 |
| 231200 | Bernard-Soulier syndrome | GP1BB | 2812 |
| 231200 | Bernard-Soulier syndrome | GP9 | 2815 |
| 231680 | Glutaric acidemia IIA | ETFA | 2108 |
| 231680 | Glutaric acidemia IIA | ETFB | 2109 |
| 231680 | Glutaric acidemia IIA | ETFDH | 2110 |
| 235400 | Hemolytic uremic syndrome | CFH | 3075 |
| 235400 | Hemolytic uremic syndrome | CFHR1 | 3078 |
| 235400 | Hemolytic uremic syndrome | CFHR3 | 10878 |
| 242600 | Iminoglycinuria | SLC6A20 | 54716 |
| 242600 | Iminoglycinuria | SLC36A2 | 153201 |
| 242600 | Iminoglycinuria | SLC6A19 | 340024 |
| 248600 | Maple syrup urine disease | BCKDHA | 593 |
| 248600 | Maple syrup urine disease | BCKDHB | 594 |
| 248600 | Maple syrup urine disease | DBT | 1629 |
| 253290 | Multiple pterygium syndrome | CHRNA1 | 1134 |
| 253290 | Multiple pterygium syndrome | CHRND | 1144 |
| 253290 | Multiple pterygium syndrome | CHRNG | 1146 |
| 254090 | Ullrich congenital muscular dystrophy 1 | COL6A1 | 1291 |
| 254090 | Ullrich congenital muscular dystrophy 1 | COL6A2 | 1292 |
| 254090 | Ullrich congenital muscular dystrophy 1 | COL6A3 | 1293 |
| 255310 | Myopathy | ACTA1 | 58 |
| 255310 | Myopathy | TPM3 | 7170 |
| 255310 | Myopathy | SEPN1 | 57190 |
| 259500 | Osteosarcoma | RB1 | 5925 |
| 259500 | Osteosarcoma | TP53 | 7157 |
| 259500 | Osteosarcoma | CHEK2 | 11200 |
| 264350 | Pseudohypoaldosteronism | SCNN1A | 6337 |
| 264350 | Pseudohypoaldosteronism | SCNN1B | 6338 |
| 264350 | Pseudohypoaldosteronism | SCNN1G | 6340 |
| 264800 | Pseudoxanthoma elasticum | ABCC6 | 368 |
| 264800 | Pseudoxanthoma elasticum | XYLT1 | 64131 |
| 264800 | Pseudoxanthoma elasticum | XYLT2 | 64132 |
| 268220 | Rhabdomyosarcoma 2 | FOXO1 | 2308 |
| 268220 | Rhabdomyosarcoma 2 | PAX3 | 5077 |
| 268220 | Rhabdomyosarcoma 2 | PAX7 | 5081 |
| 269160 | Schizencephaly | EMX2 | 2018 |
| 269160 | Schizencephaly | SHH | 6469 |
| 269160 | Schizencephaly | SIX3 | 6496 |
| 273300 | Germ cell tumors | FGFR3 | 2261 |
| 273300 | Germ cell tumors | KIT | 3815 |
| 273300 | Germ cell tumors | STK11 | 6794 |
| 600791 | Deafness | FOXI1 | 2299 |
| 600791 | Deafness | KCNJ10 | 3766 |
| 600791 | Deafness | SLC26A4 | 5172 |
| 602089 | Hemangioma | FLT4 | 2324 |
| 602089 | Hemangioma | KDR | 3791 |
| 602089 | Hemangioma | ANTXR1 | 84168 |
| 603554 | Omenn syndrome | RAG1 | 5896 |
| 603554 | Omenn syndrome | RAG2 | 5897 |
| 603554 | Omenn syndrome | DCLRE1C | 64421 |
| 604229 | Peters anomaly | CYP1B1 | 1545 |
| 604229 | Peters anomaly | PAX6 | 5080 |
| 604229 | Peters anomaly | PITX2 | 5308 |
| 604571 | Bare lymphocyte syndrome | TAP1 | 6890 |
| 604571 | Bare lymphocyte syndrome | TAP2 | 6891 |
| 604571 | Bare lymphocyte syndrome | TAPBP | 6892 |
| 605462 | Basal cell carcinoma | PTCH1 | 5727 |
| 605462 | Basal cell carcinoma | RASA1 | 5921 |
| 605462 | Basal cell carcinoma | PTCH2 | 8643 |
| 605899 | Glycine encephalopathy | AMT | 275 |
| 605899 | Glycine encephalopathy | GCSH | 2653 |
| 605899 | Glycine encephalopathy | GLDC | 2731 |
| 606864 | Paraganglioma and gastric stromal sarcoma | SDHB | 6390 |
| 606864 | Paraganglioma and gastric stromal sarcoma | SDHC | 6391 |
| 606864 | Paraganglioma and gastric stromal sarcoma | SDHD | 6392 |
| 606963 | Pulmonary disease | HMOX1 | 3162 |
| 606963 | Pulmonary disease | MMP1 | 4312 |
| 606963 | Pulmonary disease | SERPINA1 | 5265 |
| 607748 | Hypercholanemia | BAAT | 570 |
| 607748 | Hypercholanemia | EPHX1 | 2052 |
| 607748 | Hypercholanemia | TJP2 | 9414 |
| 608516 | Major depressive disorder | FKBP5 | 2289 |
| 608516 | Major depressive disorder | HTR2A | 3356 |
| 608516 | Major depressive disorder | TPH2 | 121278 |

Table S2. The 572 disease genes corresponding to the 54 diseases which are included in the DisGeNET database.

| OMIM ID | Phenotype | Gene symbol | Entrez ID |
| --- | --- | --- | --- |
| 125853 | Diabetes mellitus | CP | 1356 |
| 125853 | Diabetes mellitus | GLIS3 | 169792 |
| 125853 | Diabetes mellitus | PTPN22 | 26191 |
| 125853 | Diabetes mellitus | ZFP57 | 346171 |
| 125853 | Diabetes mellitus | AQP2 | 359 |
| 125853 | Diabetes mellitus | INS | 3630 |
| 125853 | Diabetes mellitus | INSR | 3643 |
| 125853 | Diabetes mellitus | FOXP3 | 50943 |
| 125853 | Diabetes mellitus | IER3IP1 | 51124 |
| 125853 | Diabetes mellitus | PLAGL1 | 5325 |
| 125853 | Diabetes mellitus | PPP1R3A | 5506 |
| 125853 | Diabetes mellitus | AVP | 551 |
| 125853 | Diabetes mellitus | AVPR2 | 554 |
| 125853 | Diabetes mellitus | HYMAI | 57061 |
| 125853 | Diabetes mellitus | SLC2A4 | 6517 |
| 125853 | Diabetes mellitus | KLF11 | 8462 |
| 114500 | Colon cancer | MLH1 | 4292 |
| 114480 | Breast cancer | PRLR | 5618 |
| 114480 | Breast cancer | RAD51C | 5889 |
| 114480 | Breast cancer | BRCA1 | 672 |
| 601626 | Leukemia | ARL11 | 115761 |
| 601626 | Leukemia | ARHGAP26 | 23092 |
| 601626 | Leukemia | ABL1 | 25 |
| 601626 | Leukemia | SETBP1 | 26040 |
| 601626 | Leukemia | IGHV3-21 | 28444 |
| 601626 | Leukemia | KMT2A | 4297 |
| 601626 | Leukemia | MYH11 | 4629 |
| 601626 | Leukemia | ATM | 472 |
| 601626 | Leukemia | NF1 | 4763 |
| 601626 | Leukemia | NRAS | 4893 |
| 601626 | Leukemia | NUMA1 | 4926 |
| 601626 | Leukemia | P2RX7 | 5027 |
| 601626 | Leukemia | PAX5 | 5079 |
| 601626 | Leukemia | PML | 5371 |
| 601626 | Leukemia | PRKAR1A | 5573 |
| 601626 | Leukemia | PTPN11 | 5781 |
| 601626 | Leukemia | RARA | 5914 |
| 601626 | Leukemia | CCND1 | 595 |
| 601626 | Leukemia | BCR | 613 |
| 601626 | Leukemia | TP53 | 7157 |
| 601626 | Leukemia | ZBTB16 | 7704 |
| 601626 | Leukemia | CBL | 867 |
| 256000 | Leigh syndrome | LRPPRC | 10128 |
| 256000 | Leigh syndrome | ATP6 | 4508 |
| 181500 | Schizophrenia | GJA5 | 2702 |
| 181500 | Schizophrenia | GJA8 | 2703 |
| 181500 | Schizophrenia | DISC2 | 27184 |
| 181500 | Schizophrenia | NRG1 | 3084 |
| 181500 | Schizophrenia | ATP2A2 | 488 |
| 181500 | Schizophrenia | MAGI2 | 9863 |
| 601665 | Obesity | RAI1 | 10743 |
| 601665 | Obesity | DPYD | 1806 |
| 601665 | Obesity | SH2B1 | 25970 |
| 601665 | Obesity | AFF4 | 27125 |
| 601665 | Obesity | GNAS | 2778 |
| 601665 | Obesity | APOE | 348 |
| 601665 | Obesity | LEP | 3952 |
| 601665 | Obesity | LEPR | 3953 |
| 601665 | Obesity | NTRK2 | 4915 |
| 601665 | Obesity | PCSK1 | 5122 |
| 608446 | Myocardial infarcation | SH2B3 | 10019 |
| 608446 | Myocardial infarcation | ACE | 1636 |
| 176807 | Prostate cancer | HOXB13 | 10481 |
| 176807 | Prostate cancer | RNASEL | 6041 |
| 104300 | Alzheimer disease | APOE | 348 |
| 104300 | Alzheimer disease | PSEN2 | 5664 |
| 600807 | Asthma | TBX21 | 30009 |
| 600807 | Asthma | IL4 | 3565 |
| 180300 | Rheumatoid arthritis | IL6 | 3569 |
| 180300 | Rheumatoid arthritis | MIF | 4282 |
| 180300 | Rheumatoid arthritis | TNF | 7124 |
| 133239 | Esophageal cancer | RHBDF2 | 79651 |
| 143890 | Hypercholesterolemia | CYP7A1 | 1581 |
| 143890 | Hypercholesterolemia | PCSK9 | 255738 |
| 143890 | Hypercholesterolemia | LDLRAP1 | 26119 |
| 143890 | Hypercholesterolemia | APOB | 338 |
| 143890 | Hypercholesterolemia | APOE | 348 |
| 143890 | Hypercholesterolemia | LPL | 4023 |
| 144700 | Renal cell carcinoma | MET | 4233 |
| 144700 | Renal cell carcinoma | PIK3CA | 5290 |
| 144700 | Renal cell carcinoma | TFE3 | 7030 |
| 144700 | Renal cell carcinoma | TP53 | 7157 |
| 144700 | Renal cell carcinoma | TSC1 | 7248 |
| 187500 | Tetralogy of Fallot | CITED2 | 10370 |
| 187500 | Tetralogy of Fallot | FOXC2 | 2303 |
| 187500 | Tetralogy of Fallot | GJA5 | 2702 |
| 166710 | Osteoporosis | CYP19A1 | 1588 |
| 166710 | Osteoporosis | TNFRSF11B | 4982 |
| 166710 | Osteoporosis | SLC34A1 | 6569 |
| 166710 | Osteoporosis | WNT1 | 7471 |
| 166710 | Osteoporosis | SLC9A3R1 | 9368 |
| 607174 | Meningioma | AKT1 | 207 |
| 607174 | Meningioma | BAP1 | 8314 |
| 167800 | Pancreatitis | LPL | 4023 |
| 168600 | Pancreatitis | PARK7 | 11315 |
| 168600 | Pancreatitis | LRRK2 | 120892 |
| 168600 | Pancreatitis | FBXO7 | 25793 |
| 168600 | Pancreatitis | GIGYF2 | 26058 |
| 168600 | Pancreatitis | HTRA2 | 27429 |
| 168600 | Pancreatitis | PARK2 | 5071 |
| 168600 | Pancreatitis | PINK1 | 65018 |
| 168600 | Pancreatitis | SNCA | 6622 |
| 168600 | Pancreatitis | UCHL1 | 7345 |
| 168600 | Pancreatitis | PLA2G6 | 8398 |
| 222100 | Diabetes, type 1 | CP | 1356 |
| 222100 | Diabetes, type 1 | GLIS3 | 169792 |
| 222100 | Diabetes, type 1 | AKT2 | 208 |
| 222100 | Diabetes, type 1 | GCK | 2645 |
| 222100 | Diabetes, type 1 | HNF4A | 3172 |
| 222100 | Diabetes, type 1 | ZFP57 | 346171 |
| 222100 | Diabetes, type 1 | AQP2 | 359 |
| 222100 | Diabetes, type 1 | INS | 3630 |
| 222100 | Diabetes, type 1 | INSR | 3643 |
| 222100 | Diabetes, type 1 | PDX1 | 3651 |
| 222100 | Diabetes, type 1 | IRS1 | 3667 |
| 222100 | Diabetes, type 1 | KCNJ11 | 3767 |
| 222100 | Diabetes, type 1 | TRNL1 | 4567 |
| 222100 | Diabetes, type 1 | NEUROD1 | 4760 |
| 222100 | Diabetes, type 1 | PAX4 | 5078 |
| 222100 | Diabetes, type 1 | IER3IP1 | 51124 |
| 222100 | Diabetes, type 1 | PLAGL1 | 5325 |
| 222100 | Diabetes, type 1 | PPARG | 5468 |
| 222100 | Diabetes, type 1 | PPP1R3A | 5506 |
| 222100 | Diabetes, type 1 | AVP | 551 |
| 222100 | Diabetes, type 1 | AVPR2 | 554 |
| 222100 | Diabetes, type 1 | HYMAI | 57061 |
| 222100 | Diabetes, type 1 | SLC2A4 | 6517 |
| 222100 | Diabetes, type 1 | ABCC8 | 6833 |
| 222100 | Diabetes, type 1 | HNF1B | 6928 |
| 222100 | Diabetes, type 1 | KLF11 | 8462 |
| 222100 | Diabetes, type 1 | MAPK8IP1 | 9479 |
| 226650 | Epidermolysis bullosa | COL7A1 | 1294 |
| 226650 | Epidermolysis bullosa | DSP | 1832 |
| 226650 | Epidermolysis bullosa | ITGA6 | 3655 |
| 226650 | Epidermolysis bullosa | ITGA3 | 3675 |
| 226650 | Epidermolysis bullosa | KRT5 | 3852 |
| 226650 | Epidermolysis bullosa | KRT14 | 3861 |
| 226650 | Epidermolysis bullosa | PLEC | 5339 |
| 226650 | Epidermolysis bullosa | CD151 | 977 |
| 603896 | Leukoencephalopathy with vanishing white matter | CLCN2 | 1181 |
| 603896 | Leukoencephalopathy with vanishing white matter | CSF1R | 1436 |
| 603896 | Leukoencephalopathy with vanishing white matter | MLC1 | 23209 |
| 603896 | Leukoencephalopathy with vanishing white matter | TREM2 | 54209 |
| 603896 | Leukoencephalopathy with vanishing white matter | DARS2 | 55157 |
| 603896 | Leukoencephalopathy with vanishing white matter | HTRA1 | 5654 |
| 603896 | Leukoencephalopathy with vanishing white matter | SCP2 | 6342 |
| 603896 | Leukoencephalopathy with vanishing white matter | TYROBP | 7305 |
| 603896 | Leukoencephalopathy with vanishing white matter | RNASET2 | 8635 |
| 605027 | Lymphoma, follicular | CTLA4 | 1493 |
| 605027 | Lymphoma, follicular | MLH1 | 4292 |
| 605027 | Lymphoma, follicular | MYC | 4609 |
| 605027 | Lymphoma, follicular | ATM | 472 |
| 605027 | Lymphoma, follicular | NRAS | 4893 |
| 605027 | Lymphoma, follicular | PAX5 | 5079 |
| 605027 | Lymphoma, follicular | CCND1 | 595 |
| 605027 | Lymphoma, follicular | BCL2 | 596 |
| 605027 | Lymphoma, follicular | CD28 | 940 |
| 105200 | Amyloidosis, familial visceral | APP | 351 |
| 105200 | Amyloidosis, familial visceral | TTR | 7276 |
| 105200 | Amyloidosis, familial visceral | OSMR | 9180 |
| 105400 | Amyotrophic lateral sclerosis | OPTN | 10133 |
| 105400 | Amyotrophic lateral sclerosis | SIGMAR1 | 10280 |
| 105400 | Amyotrophic lateral sclerosis | PPARGC1A | 10891 |
| 105400 | Amyotrophic lateral sclerosis | DAO | 1610 |
| 105400 | Amyotrophic lateral sclerosis | EPHA4 | 2043 |
| 105400 | Amyotrophic lateral sclerosis | ERBB4 | 2066 |
| 105400 | Amyotrophic lateral sclerosis | UNC13A | 23025 |
| 105400 | Amyotrophic lateral sclerosis | SETX | 23064 |
| 105400 | Amyotrophic lateral sclerosis | TARDBP | 23435 |
| 105400 | Amyotrophic lateral sclerosis | FUS | 2521 |
| 105400 | Amyotrophic lateral sclerosis | CHMP2B | 25978 |
| 105400 | Amyotrophic lateral sclerosis | ANG | 283 |
| 105400 | Amyotrophic lateral sclerosis | TBK1 | 29110 |
| 105400 | Amyotrophic lateral sclerosis | UBQLN2 | 29978 |
| 105400 | Amyotrophic lateral sclerosis | HNRNPA1 | 3178 |
| 105400 | Amyotrophic lateral sclerosis | PFN1 | 5216 |
| 105400 | Amyotrophic lateral sclerosis | TREM2 | 54209 |
| 105400 | Amyotrophic lateral sclerosis | PON1 | 5444 |
| 105400 | Amyotrophic lateral sclerosis | PON2 | 5445 |
| 105400 | Amyotrophic lateral sclerosis | PON3 | 5446 |
| 105400 | Amyotrophic lateral sclerosis | TRPM7 | 54822 |
| 105400 | Amyotrophic lateral sclerosis | ATXN2 | 6311 |
| 105400 | Amyotrophic lateral sclerosis | VCP | 7415 |
| 105400 | Amyotrophic lateral sclerosis | SQSTM1 | 8878 |
| 105400 | Amyotrophic lateral sclerosis | VAPB | 9217 |
| 105400 | Amyotrophic lateral sclerosis | MATR3 | 9782 |
| 105400 | Amyotrophic lateral sclerosis | FIG4 | 9896 |
| 162900 | Dejerine-Sottas disease | KRAS | 3845 |
| 188050 | Thromboembolism | JAK2 | 3717 |
| 209920 | Bare lymphocyte syndrome | TAP1 | 6890 |
| 209920 | Bare lymphocyte syndrome | TAP2 | 6891 |
| 209920 | Bare lymphocyte syndrome | TAPBP | 6892 |
| 260350 | Pancreatic cancer | CDKN2A | 1029 |
| 601680 | Arthrogryposis multiplex congenita | CHRNG | 1146 |
| 601680 | Arthrogryposis multiplex congenita | ERCC6 | 2074 |
| 601680 | Arthrogryposis multiplex congenita | VPS33B | 26276 |
| 601680 | Arthrogryposis multiplex congenita | GLE1 | 2733 |
| 601680 | Arthrogryposis multiplex congenita | VIPAS39 | 63894 |
| 601680 | Arthrogryposis multiplex congenita | PIEZO2 | 63895 |
| 601680 | Arthrogryposis multiplex congenita | UBA1 | 7317 |
| 601680 | Arthrogryposis multiplex congenita | ECEL1 | 9427 |
| 606176 | Diabetes mellitus | CP | 1356 |
| 606176 | Diabetes mellitus | GLIS3 | 169792 |
| 606176 | Diabetes mellitus | AKT2 | 208 |
| 606176 | Diabetes mellitus | PTPN22 | 26191 |
| 606176 | Diabetes mellitus | HNF4A | 3172 |
| 606176 | Diabetes mellitus | ZFP57 | 346171 |
| 606176 | Diabetes mellitus | AQP2 | 359 |
| 606176 | Diabetes mellitus | INSR | 3643 |
| 606176 | Diabetes mellitus | PDX1 | 3651 |
| 606176 | Diabetes mellitus | IRS1 | 3667 |
| 606176 | Diabetes mellitus | NEUROD1 | 4760 |
| 606176 | Diabetes mellitus | PAX4 | 5078 |
| 606176 | Diabetes mellitus | FOXP3 | 50943 |
| 606176 | Diabetes mellitus | IER3IP1 | 51124 |
| 606176 | Diabetes mellitus | PLAGL1 | 5325 |
| 606176 | Diabetes mellitus | PPARG | 5468 |
| 606176 | Diabetes mellitus | PPP1R3A | 5506 |
| 606176 | Diabetes mellitus | AVP | 551 |
| 606176 | Diabetes mellitus | AVPR2 | 554 |
| 606176 | Diabetes mellitus | HYMAI | 57061 |
| 606176 | Diabetes mellitus | SLC2A4 | 6517 |
| 606176 | Diabetes mellitus | HNF1A | 6927 |
| 606176 | Diabetes mellitus | HNF1B | 6928 |
| 606176 | Diabetes mellitus | KLF11 | 8462 |
| 606176 | Diabetes mellitus | MAPK8IP1 | 9479 |
| 606764 | Gastrointestinal stromal tumor | SDHA | 6389 |
| 607785 | Leukemia | CEBPA | 1050 |
| 607785 | Leukemia | ARL11 | 115761 |
| 607785 | Leukemia | FLT3 | 2322 |
| 607785 | Leukemia | ABL1 | 25 |
| 607785 | Leukemia | SETBP1 | 26040 |
| 607785 | Leukemia | IGHV3-21 | 28444 |
| 607785 | Leukemia | JAK2 | 3717 |
| 607785 | Leukemia | KIT | 3815 |
| 607785 | Leukemia | KRAS | 3845 |
| 607785 | Leukemia | KMT2A | 4297 |
| 607785 | Leukemia | MYH11 | 4629 |
| 607785 | Leukemia | ATM | 472 |
| 607785 | Leukemia | NPM1 | 4869 |
| 607785 | Leukemia | NRAS | 4893 |
| 607785 | Leukemia | NUMA1 | 4926 |
| 607785 | Leukemia | P2RX7 | 5027 |
| 607785 | Leukemia | PAX5 | 5079 |
| 607785 | Leukemia | PML | 5371 |
| 607785 | Leukemia | PRKAR1A | 5573 |
| 607785 | Leukemia | RARA | 5914 |
| 607785 | Leukemia | CCND1 | 595 |
| 607785 | Leukemia | BCR | 613 |
| 607785 | Leukemia | TP53 | 7157 |
| 607785 | Leukemia | ZBTB16 | 7704 |
| 607785 | Leukemia | RUNX1 | 861 |
| 607785 | Leukemia | CBFB | 865 |
| 607785 | Leukemia | CBL | 867 |
| 112600 | Brachydactyly | CHSY1 | 22856 |
| 112600 | Brachydactyly | GNAS | 2778 |
| 112600 | Brachydactyly | HOXD13 | 3239 |
| 112600 | Brachydactyly | IHH | 3549 |
| 112600 | Brachydactyly | SMAD4 | 4089 |
| 112600 | Brachydactyly | ROR2 | 4920 |
| 112600 | Brachydactyly | PDE3A | 5139 |
| 112600 | Brachydactyly | PTHLH | 5744 |
| 112600 | Brachydactyly | TRPV4 | 59341 |
| 112600 | Brachydactyly | NOG | 9241 |
| 112600 | Brachydactyly | HDAC4 | 9759 |
| 130000 | Ehlers-Danlos syndrome | B4GALT7 | 11285 |
| 130000 | Ehlers-Danlos syndrome | CHST14 | 113189 |
| 130000 | Ehlers-Danlos syndrome | COL1A2 | 1278 |
| 130000 | Ehlers-Danlos syndrome | COL3A1 | 1281 |
| 130000 | Ehlers-Danlos syndrome | PLOD1 | 5351 |
| 130000 | Ehlers-Danlos syndrome | TNXB | 7148 |
| 130000 | Ehlers-Danlos syndrome | C1R | 715 |
| 130000 | Ehlers-Danlos syndrome | ZNF469 | 84627 |
| 130000 | Ehlers-Danlos syndrome | SLC39A13 | 91252 |
| 130000 | Ehlers-Danlos syndrome | ADAMTS2 | 9509 |
| 133200 | Erythrokeratodermia variabilis et progressiva | AP1S1 | 1174 |
| 133200 | Erythrokeratodermia variabilis et progressiva | ELOVL4 | 6785 |
| 155255 | Medulloblastoma | CTNNB1 | 1499 |
| 155255 | Medulloblastoma | APC | 324 |
| 155255 | Medulloblastoma | PTCH1 | 5727 |
| 157300 | Migraine without aura | TGFBR2 | 7048 |
| 192600 | Cardiomyopathy | ABCC9 | 10060 |
| 192600 | Cardiomyopathy | LDB3 | 11155 |
| 192600 | Cardiomyopathy | DES | 1674 |
| 192600 | Cardiomyopathy | DMD | 1756 |
| 192600 | Cardiomyopathy | DSP | 1832 |
| 192600 | Cardiomyopathy | EYA4 | 2070 |
| 192600 | Cardiomyopathy | FKTN | 2218 |
| 192600 | Cardiomyopathy | GJA5 | 2702 |
| 192600 | Cardiomyopathy | RBM20 | 282996 |
| 192600 | Cardiomyopathy | LMNA | 4000 |
| 192600 | Cardiomyopathy | CYTB | 4519 |
| 192600 | Cardiomyopathy | MYBPC3 | 4607 |
| 192600 | Cardiomyopathy | MYH6 | 4624 |
| 192600 | Cardiomyopathy | MYL2 | 4633 |
| 192600 | Cardiomyopathy | MYL3 | 4634 |
| 192600 | Cardiomyopathy | NDUFS1 | 4719 |
| 192600 | Cardiomyopathy | TNNI3K | 51086 |
| 192600 | Cardiomyopathy | PRKAG2 | 51422 |
| 192600 | Cardiomyopathy | PLN | 5350 |
| 192600 | Cardiomyopathy | TMEM70 | 54968 |
| 192600 | Cardiomyopathy | AGK | 55750 |
| 192600 | Cardiomyopathy | PSEN1 | 5663 |
| 192600 | Cardiomyopathy | PSEN2 | 5664 |
| 192600 | Cardiomyopathy | RAF1 | 5894 |
| 192600 | Cardiomyopathy | SCN5A | 6331 |
| 192600 | Cardiomyopathy | SDHA | 6389 |
| 192600 | Cardiomyopathy | SGCD | 6444 |
| 192600 | Cardiomyopathy | ACTC1 | 70 |
| 192600 | Cardiomyopathy | TMPO | 7112 |
| 192600 | Cardiomyopathy | TNNC1 | 7134 |
| 192600 | Cardiomyopathy | TNNI3 | 7137 |
| 192600 | Cardiomyopathy | TNNT2 | 7139 |
| 192600 | Cardiomyopathy | TPM1 | 7168 |
| 192600 | Cardiomyopathy | TTN | 7273 |
| 192600 | Cardiomyopathy | VCL | 7414 |
| 192600 | Cardiomyopathy | ALMS1 | 7840 |
| 192600 | Cardiomyopathy | CSRP3 | 8048 |
| 192600 | Cardiomyopathy | ACTN2 | 88 |
| 192600 | Cardiomyopathy | NEXN | 91624 |
| 192600 | Cardiomyopathy | SCO2 | 9997 |
| 194070 | Wilms tumor | POU6F2 | 11281 |
| 194070 | Wilms tumor | DIS3L2 | 129563 |
| 211900 | Tumoral calcinosis | SAMD9 | 54809 |
| 220290 | Deafness | TRIOBP | 11078 |
| 220290 | Deafness | TMC1 | 117531 |
| 220290 | Deafness | COL2A1 | 1280 |
| 220290 | Deafness | COL11A2 | 1302 |
| 220290 | Deafness | COCH | 1690 |
| 220290 | Deafness | DNMT1 | 1786 |
| 220290 | Deafness | DSPP | 1834 |
| 220290 | Deafness | AK2 | 204 |
| 220290 | Deafness | EYA4 | 2070 |
| 220290 | Deafness | ESRRB | 2103 |
| 220290 | Deafness | LRTOMT | 220074 |
| 220290 | Deafness | LHFPL5 | 222662 |
| 220290 | Deafness | FGF3 | 2248 |
| 220290 | Deafness | CLDN14 | 23562 |
| 220290 | Deafness | SLC17A8 | 246213 |
| 220290 | Deafness | MSRB3 | 253827 |
| 220290 | Deafness | TMIE | 259236 |
| 220290 | Deafness | RAB40AL | 282808 |
| 220290 | Deafness | ILDR1 | 286676 |
| 220290 | Deafness | HSD17B4 | 3295 |
| 220290 | Deafness | PTPRQ | 374462 |
| 220290 | Deafness | GRXCR1 | 389207 |
| 220290 | Deafness | SMAD4 | 4089 |
| 220290 | Deafness | TRNL1 | 4567 |
| 220290 | Deafness | MYH9 | 4627 |
| 220290 | Deafness | MYO6 | 4646 |
| 220290 | Deafness | MYO7A | 4647 |
| 220290 | Deafness | DFNB59 | 494513 |
| 220290 | Deafness | PAX3 | 5077 |
| 220290 | Deafness | MYO15A | 51168 |
| 220290 | Deafness | SLC26A4 | 5172 |
| 220290 | Deafness | PEX1 | 5189 |
| 220290 | Deafness | ATP6V1B1 | 525 |
| 220290 | Deafness | POU4F3 | 5459 |
| 220290 | Deafness | SLC29A3 | 55315 |
| 220290 | Deafness | TRMU | 55687 |
| 220290 | Deafness | PRPS1 | 5631 |
| 220290 | Deafness | RDX | 5962 |
| 220290 | Deafness | RPGR | 6103 |
| 220290 | Deafness | BCS1L | 617 |
| 220290 | Deafness | CDH23 | 64072 |
| 220290 | Deafness | TMPRSS3 | 64699 |
| 220290 | Deafness | PCDH15 | 65217 |
| 220290 | Deafness | MAP3K7 | 6885 |
| 220290 | Deafness | TECTA | 7007 |
| 220290 | Deafness | ACTG1 | 71 |
| 220290 | Deafness | WFS1 | 7466 |
| 220290 | Deafness | CACNA1D | 776 |
| 220290 | Deafness | MYH14 | 79784 |
| 220290 | Deafness | ESPN | 83715 |
| 220290 | Deafness | SLC4A11 | 83959 |
| 220290 | Deafness | SERAC1 | 84947 |
| 220290 | Deafness | PLOD3 | 8985 |
| 220290 | Deafness | AIFM1 | 9131 |
| 220290 | Deafness | KCNQ4 | 9132 |
| 220290 | Deafness | OTOF | 9381 |
| 220290 | Deafness | CD151 | 977 |
| 226700 | Epidermolysis bullosa | COL7A1 | 1294 |
| 226700 | Epidermolysis bullosa | COL17A1 | 1308 |
| 226700 | Epidermolysis bullosa | DSP | 1832 |
| 226700 | Epidermolysis bullosa | ITGA6 | 3655 |
| 226700 | Epidermolysis bullosa | ITGA3 | 3675 |
| 226700 | Epidermolysis bullosa | ITGB4 | 3691 |
| 226700 | Epidermolysis bullosa | KRT5 | 3852 |
| 226700 | Epidermolysis bullosa | KRT14 | 3861 |
| 226700 | Epidermolysis bullosa | PLEC | 5339 |
| 226700 | Epidermolysis bullosa | CD151 | 977 |
| 235400 | Ullrich congenital muscular dystrophy 1 | CFI | 3426 |
| 235400 | Ullrich congenital muscular dystrophy 1 | CD46 | 4179 |
| 235400 | Ullrich congenital muscular dystrophy 1 | CFB | 629 |
| 235400 | Ullrich congenital muscular dystrophy 1 | THBD | 7056 |
| 235400 | Ullrich congenital muscular dystrophy 1 | C3 | 718 |
| 253290 | Multiple pterygium syndrome | MYH3 | 4621 |
| 255310 | Myopathy | GNE | 10020 |
| 255310 | Myopathy | ABCC9 | 10060 |
| 255310 | Myopathy | RBCK1 | 10616 |
| 255310 | Myopathy | CFL2 | 1073 |
| 255310 | Myopathy | LDB3 | 11155 |
| 255310 | Myopathy | CNTN1 | 1272 |
| 255310 | Myopathy | COL6A1 | 1291 |
| 255310 | Myopathy | COL6A2 | 1292 |
| 255310 | Myopathy | COL6A3 | 1293 |
| 255310 | Myopathy | CPT2 | 1376 |
| 255310 | Myopathy | DES | 1674 |
| 255310 | Myopathy | DMD | 1756 |
| 255310 | Myopathy | DNM2 | 1785 |
| 255310 | Myopathy | DSP | 1832 |
| 255310 | Myopathy | VMA21 | 203547 |
| 255310 | Myopathy | EYA4 | 2070 |
| 255310 | Myopathy | FKTN | 2218 |
| 255310 | Myopathy | FHL1 | 2273 |
| 255310 | Myopathy | FLNC | 2318 |
| 255310 | Myopathy | ISCU | 23479 |
| 255310 | Myopathy | STAC3 | 246329 |
| 255310 | Myopathy | GFER | 2671 |
| 255310 | Myopathy | GJA5 | 2702 |
| 255310 | Myopathy | BIN1 | 274 |
| 255310 | Myopathy | RBM20 | 282996 |
| 255310 | Myopathy | GYG1 | 2992 |
| 255310 | Myopathy | HADHA | 3030 |
| 255310 | Myopathy | HADHB | 3032 |
| 255310 | Myopathy | FAM111B | 374393 |
| 255310 | Myopathy | KBTBD13 | 390594 |
| 255310 | Myopathy | LMNA | 4000 |
| 255310 | Myopathy | CHCHD10 | 400916 |
| 255310 | Myopathy | CYTB | 4519 |
| 255310 | Myopathy | MTM1 | 4534 |
| 255310 | Myopathy | RNR1 | 4549 |
| 255310 | Myopathy | TRNT | 4576 |
| 255310 | Myopathy | MYBPC3 | 4607 |
| 255310 | Myopathy | MYF6 | 4618 |
| 255310 | Myopathy | MYH2 | 4620 |
| 255310 | Myopathy | MYH6 | 4624 |
| 255310 | Myopathy | MYH7 | 4625 |
| 255310 | Myopathy | MYL2 | 4633 |
| 255310 | Myopathy | MYL3 | 4634 |
| 255310 | Myopathy | NDUFS1 | 4719 |
| 255310 | Myopathy | ATP2A1 | 487 |
| 255310 | Myopathy | YARS2 | 51067 |
| 255310 | Myopathy | TNNI3K | 51086 |
| 255310 | Myopathy | PRKAG2 | 51422 |
| 255310 | Myopathy | PLN | 5350 |
| 255310 | Myopathy | TMEM70 | 54968 |
| 255310 | Myopathy | AGK | 55750 |
| 255310 | Myopathy | PSEN1 | 5663 |
| 255310 | Myopathy | PSEN2 | 5664 |
| 255310 | Myopathy | PNPLA2 | 57104 |
| 255310 | Myopathy | PYGM | 5837 |
| 255310 | Myopathy | RAF1 | 5894 |
| 255310 | Myopathy | RYR1 | 6261 |
| 255310 | Myopathy | SCN5A | 6331 |
| 255310 | Myopathy | SDHA | 6389 |
| 255310 | Myopathy | MTMR14 | 64419 |
| 255310 | Myopathy | SGCD | 6444 |
| 255310 | Myopathy | ACTC1 | 70 |
| 255310 | Myopathy | TMPO | 7112 |
| 255310 | Myopathy | TNNC1 | 7134 |
| 255310 | Myopathy | TNNI3 | 7137 |
| 255310 | Myopathy | TNNT1 | 7138 |
| 255310 | Myopathy | TNNT2 | 7139 |
| 255310 | Myopathy | TPM1 | 7168 |
| 255310 | Myopathy | TPM2 | 7169 |
| 255310 | Myopathy | ACTG2 | 72 |
| 255310 | Myopathy | TTN | 7273 |
| 255310 | Myopathy | VCL | 7414 |
| 255310 | Myopathy | VCP | 7415 |
| 255310 | Myopathy | ALMS1 | 7840 |
| 255310 | Myopathy | MYH14 | 79784 |
| 255310 | Myopathy | PUS1 | 80324 |
| 255310 | Myopathy | CSRP3 | 8048 |
| 255310 | Myopathy | DYSF | 8291 |
| 255310 | Myopathy | CASQ1 | 844 |
| 255310 | Myopathy | MEGF10 | 84466 |
| 255310 | Myopathy | MYLK2 | 85366 |
| 255310 | Myopathy | CAV3 | 859 |
| 255310 | Myopathy | ACTN2 | 88 |
| 255310 | Myopathy | NEXN | 91624 |
| 255310 | Myopathy | MYOT | 9499 |
| 255310 | Myopathy | BAG3 | 9531 |
| 255310 | Myopathy | SCO2 | 9997 |
| 264800 | Pseudoxanthoma elasticum | GGCX | 2677 |
| 269160 | Schizencephaly | COL4A1 | 1282 |
| 600791 | Deafness | GJB6 | 10804 |
| 600791 | Deafness | TRIOBP | 11078 |
| 600791 | Deafness | TMC1 | 117531 |
| 600791 | Deafness | COL2A1 | 1280 |
| 600791 | Deafness | COL11A2 | 1302 |
| 600791 | Deafness | COCH | 1690 |
| 600791 | Deafness | DNMT1 | 1786 |
| 600791 | Deafness | DSPP | 1834 |
| 600791 | Deafness | AK2 | 204 |
| 600791 | Deafness | EYA4 | 2070 |
| 600791 | Deafness | ESRRB | 2103 |
| 600791 | Deafness | LRTOMT | 220074 |
| 600791 | Deafness | LHFPL5 | 222662 |
| 600791 | Deafness | FGF3 | 2248 |
| 600791 | Deafness | CLDN14 | 23562 |
| 600791 | Deafness | SLC17A8 | 246213 |
| 600791 | Deafness | MSRB3 | 253827 |
| 600791 | Deafness | TMIE | 259236 |
| 600791 | Deafness | GJB2 | 2706 |
| 600791 | Deafness | GJB3 | 2707 |
| 600791 | Deafness | RAB40AL | 282808 |
| 600791 | Deafness | ILDR1 | 286676 |
| 600791 | Deafness | HSD17B4 | 3295 |
| 600791 | Deafness | PTPRQ | 374462 |
| 600791 | Deafness | GRXCR1 | 389207 |
| 600791 | Deafness | SMAD4 | 4089 |
| 600791 | Deafness | TRNL1 | 4567 |
| 600791 | Deafness | MYH9 | 4627 |
| 600791 | Deafness | MYO6 | 4646 |
| 600791 | Deafness | MYO7A | 4647 |
| 600791 | Deafness | DFNB59 | 494513 |
| 600791 | Deafness | PAX3 | 5077 |
| 600791 | Deafness | MYO15A | 51168 |
| 600791 | Deafness | PEX1 | 5189 |
| 600791 | Deafness | ATP6V1B1 | 525 |
| 600791 | Deafness | POU4F3 | 5459 |
| 600791 | Deafness | SLC29A3 | 55315 |
| 600791 | Deafness | TRMU | 55687 |
| 600791 | Deafness | PRPS1 | 5631 |
| 600791 | Deafness | RDX | 5962 |
| 600791 | Deafness | RPGR | 6103 |
| 600791 | Deafness | BCS1L | 617 |
| 600791 | Deafness | CDH23 | 64072 |
| 600791 | Deafness | TMPRSS3 | 64699 |
| 600791 | Deafness | PCDH15 | 65217 |
| 600791 | Deafness | MAP3K7 | 6885 |
| 600791 | Deafness | TECTA | 7007 |
| 600791 | Deafness | ACTG1 | 71 |
| 600791 | Deafness | WFS1 | 7466 |
| 600791 | Deafness | CACNA1D | 776 |
| 600791 | Deafness | MYH14 | 79784 |
| 600791 | Deafness | ESPN | 83715 |
| 600791 | Deafness | SLC4A11 | 83959 |
| 600791 | Deafness | SERAC1 | 84947 |
| 600791 | Deafness | PLOD3 | 8985 |
| 600791 | Deafness | AIFM1 | 9131 |
| 600791 | Deafness | KCNQ4 | 9132 |
| 600791 | Deafness | OTOF | 9381 |
| 600791 | Deafness | CD151 | 977 |
| 602089 | Hemangioma | PTEN | 5728 |
| 602089 | Hemangioma | KRIT1 | 889 |
| 604571 | Bare lymphocyte syndrome | CIITA | 4261 |
| 604571 | Bare lymphocyte syndrome | RFX5 | 5993 |
| 604571 | Bare lymphocyte syndrome | RFXAP | 5994 |
| 604571 | Bare lymphocyte syndrome | RFXANK | 8625 |
| 605462 | Basal cell carcinoma | CDKN2A | 1029 |
| 605462 | Basal cell carcinoma | RNF139 | 11236 |
| 605462 | Basal cell carcinoma | RSPO1 | 284654 |
| 605462 | Basal cell carcinoma | ING1 | 3621 |
| 605462 | Basal cell carcinoma | MET | 4233 |
| 605462 | Basal cell carcinoma | OGG1 | 4968 |
| 605462 | Basal cell carcinoma | SUFU | 51684 |
| 605462 | Basal cell carcinoma | PIK3CA | 5290 |
| 605462 | Basal cell carcinoma | PTEN | 5728 |
| 605462 | Basal cell carcinoma | RB1 | 5925 |
| 605462 | Basal cell carcinoma | SMO | 6608 |
| 605462 | Basal cell carcinoma | HNF1A | 6927 |
| 605462 | Basal cell carcinoma | TFE3 | 7030 |
| 605462 | Basal cell carcinoma | TP53 | 7157 |
| 605462 | Basal cell carcinoma | TSC1 | 7248 |
| 605462 | Basal cell carcinoma | VHL | 7428 |
| 608516 | Major depressive disorder | CNR2 | 1269 |
| 608516 | Major depressive disorder | CRH | 1392 |
| 608516 | Major depressive disorder | CRHR1 | 1394 |
| 608516 | Major depressive disorder | FREM3 | 166752 |
| 608516 | Major depressive disorder | DRD2 | 1813 |
| 608516 | Major depressive disorder | DUSP6 | 1848 |
| 608516 | Major depressive disorder | FGFR1 | 2260 |
| 608516 | Major depressive disorder | GAD1 | 2571 |
| 608516 | Major depressive disorder | GLO1 | 2739 |
| 608516 | Major depressive disorder | GRN | 2896 |
| 608516 | Major depressive disorder | NR3C1 | 2908 |
| 608516 | Major depressive disorder | GSK3B | 2932 |
| 608516 | Major depressive disorder | HTT | 3064 |
| 608516 | Major depressive disorder | HTR7 | 3363 |
| 608516 | Major depressive disorder | IL6 | 3569 |
| 608516 | Major depressive disorder | KCNK2 | 3776 |
| 608516 | Major depressive disorder | COX2 | 4513 |
| 608516 | Major depressive disorder | MTHFR | 4524 |
| 608516 | Major depressive disorder | ATP1A3 | 478 |
| 608516 | Major depressive disorder | NPY | 4852 |
| 608516 | Major depressive disorder | POMC | 5443 |
| 608516 | Major depressive disorder | PON1 | 5444 |
| 608516 | Major depressive disorder | PRNP | 5621 |
| 608516 | Major depressive disorder | RELN | 5649 |
| 608516 | Major depressive disorder | PSEN1 | 5663 |
| 608516 | Major depressive disorder | PTGS2 | 5743 |
| 608516 | Major depressive disorder | BDNF | 627 |
| 608516 | Major depressive disorder | S100A10 | 6281 |
| 608516 | Major depressive disorder | SLC6A4 | 6532 |
| 608516 | Major depressive disorder | SNAP25 | 6616 |
| 608516 | Major depressive disorder | SOD1 | 6647 |
| 608516 | Major depressive disorder | WFS1 | 7466 |
| 608516 | Major depressive disorder | CAT | 847 |
| 608516 | Major depressive disorder | FGF17 | 8822 |
| 608516 | Major depressive disorder | PER2 | 8864 |
| 608516 | Major depressive disorder | SGCE | 8910 |
